# Tailored chondroitin sulfate glycomimetics *via* a tunable multivalent scaffold for potentiating NGF/TrkA-induced neurogenesis†
†Electronic supplementary information (ESI) available: Detailed synthesis of CS disaccharides including ^1^H, ^13^C NMR, and mass data, general peptide synthesis, click reaction including FT-IR spectra of polyprolines and glycopeptides, procedures for CD measurements including spectra of glycopeptide **1–7**, ELISA and SPR measurements including complete kinetic parameters, computational methods including predicted glycopeptide binding sites on the NGF/TrkA complex in table, cellular assays and immunohistochemistry are described. See DOI: 10.1039/c4sc02553a



**DOI:** 10.1039/c4sc02553a

**Published:** 2014-10-15

**Authors:** Pei Liu, Liwei Chen, Jerry K. C. Toh, Yi Li Ang, Joo-Eun Jee, Jaehong Lim, Su Seong Lee, Song-Gil Lee

**Affiliations:** a Institute of Bioengineering and Nanotechnology , 31 Biopolis Way, The Nanos , Singapore 138669 , Singapore . Email: sglee@ibn.a-star.edu.sg ; Fax: +65 6478 9081 ; Tel: +65 6824 7131

## Abstract

The challenges inherent in the synthesis of large glycosaminoglycan (GAG) polysaccharides have made chemically accessible multivalent glycoligands a valuable tool in the field of GAG mimetics. However, the difficulty of positioning sulfated sugar motifs at desired sites has hindered efforts to precisely tailor their biofunctions. Here, we achieved precise orientation of sulfated disaccharide motifs by taking advantage of a structurally well-defined polyproline scaffold, and describe systematic explorations into the importance of the spatial arrangement of sulfated sugars along the scaffold backbone in designing multivalent glycoligands. Our protein binding studies demonstrate that the specific conformational display of pendant sugars is central to direct their multivalent interactions with NGF. By employing computational modeling and cellular studies, we have further applied this approach to engineer NGF-mediated signaling by regulating the NGF/TrkA complexation process, leading to enhanced neuronal differentiation and neurite outgrowth of PC12 cells. Our findings offer a promising strategy for the pinpoint engineering of GAG-mediated biological processes and a novel method of designing new therapeutic agents that are highly specific to GAG-associated disease.

## Abstract 2

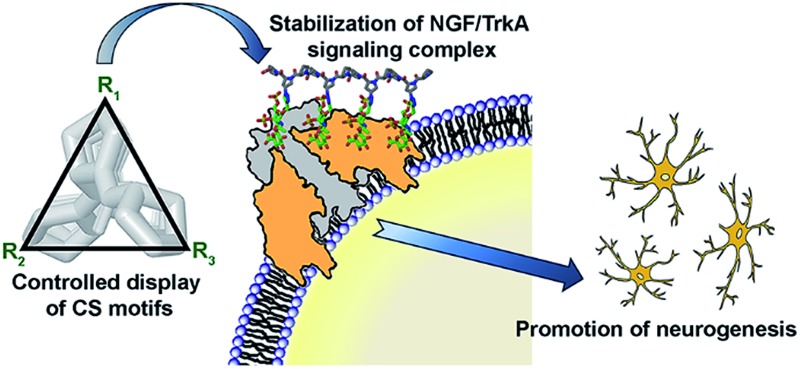
We have engineered structurally well-defined tunable chondroitin sulfate glycopeptides using a polyproline scaffold to selectively modulate the NGF-mediated neuronal signaling pathway.

## Introduction

Glycosaminoglycans (GAGs) possess enormous functional capacity and participate in many crucial physiological processes.^1^ Compared to other polysaccharides, the most prominent type of interaction between highly charged GAGs and proteins is ionic, with clusters of positively charged basic residues on protein surfaces forming ion pairs with negatively charged sulfation or carboxyl groups on GAG chains. Natural GAGs acquire protein recognition specificity by tightly regulating not only sulfation patterns, but also conformations of polysaccharide backbones in a spatiotemporal manner, conferring highly selective electrostatic interactions with proteins.^2^ Thus, accurately positioning sulfated sugars at desired sites is vital to recapitulate these specificities *in vivo* and engineer GAG-mediated signaling pathways.

Beyond chemoenzymatic methods, the most widely used mimetic strategy is to assemble multivalent architectures by conjugating bioactive sugars found in GAGs to synthetic scaffolds such as flexible linear polymers,^3^ dendrimers,^4^ and microarray surfaces.^5^ These approaches have investigated the effects of enhancing binding affinity by increasing the number of sulfated sugars or by varying the sulfation patterns of GAG moieties. While these approaches demonstrate sufficient protein binding affinity for bioactivity *in vitro*, their lack of defined spatial orientation of sulfated sugars raises the potential of nonspecific charge interactions with undesirable biocomponents, making it difficult to precisely tailor the biofunctions of GAG-based synthetic polymers.

Recently, new approaches for defined carbohydrate displays using α-helical polypeptides^6^ or foldamers^7^ as frameworks have been reported. Although the results were not directly driven from GAG moieties, these investigations highlight the importance of pre-organized arrays of pendant sugars in multivalent interactions. However, their inherent drawbacks, including variations in α-helicities and limited choice of carbohydrate displays, have thwarted efforts to accurately arrange sugars at desired positions.

Given the above challenges, in this report we describe a series of structurally well-defined glycopeptides containing chondroitin sulfate (CS) motifs and characterize their contributions towards protein recognition specificity. We demonstrate that our design strategy permits a new way to encode functional information into glycopeptides by controlling the spatial presentation of sulfated sugar motifs. We further show the efficacy of our approach to accurately engineer specific GAG-mediated physiological processes by modulating the NGF/TrkA-mediated neuronal signaling, offering a promising therapeutic strategy for the treatment of neurodegenerative disease.^8^

## Results and discussion

**Fig. 1:**
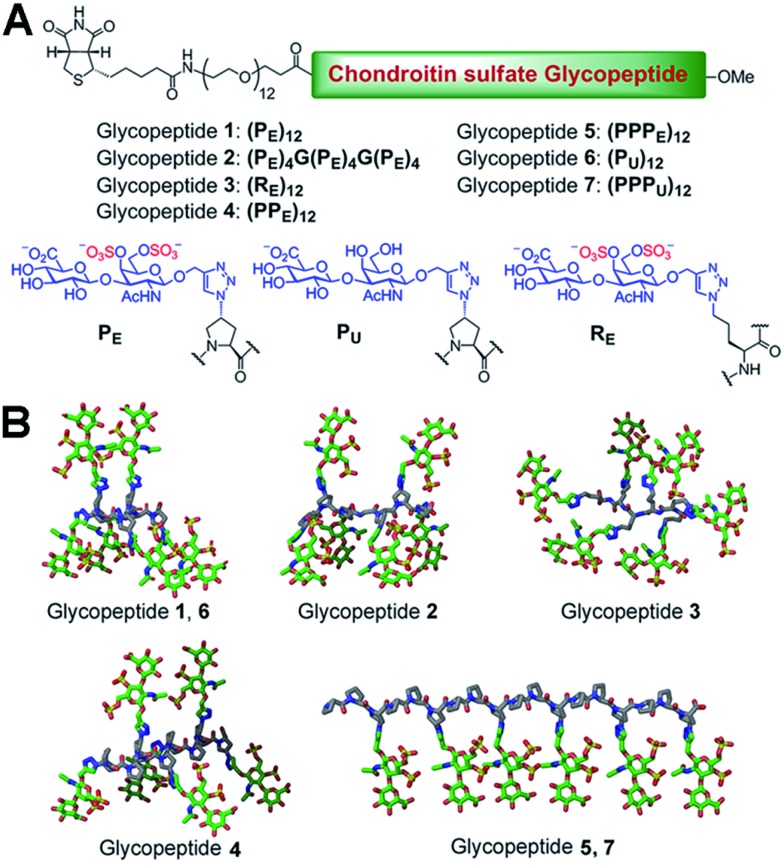
(A) Peptide sequence and structure of all glycopeptides. (B) Three-dimensional schematic showing the display of CS disaccharide units in glycopeptides **1–7**. A truncated version of the glycopeptide part is shown for greater visual clarity.

We chose a polyproline scaffold to evaluate the influence of carbohydrate display on protein recognition. A major advantage of this scaffold is to allow for the precise orientation of carbohydrate motifs at desired sites along the backbone by taking advantage of the rigid polyproline type II (PPII) helix.^9^ In our design, alkyne-bearing CS disaccharides were incorporated into the PPII helix by click reaction. A minimal distance between proline and sugar units was maintained for maximal positional control of pendant sugars. We also introduced a biotin-conjugated PEG12 chain to facilitate surface attachment (Fig. 1A).

Seven glycopeptides (Fig. 1A and B) were hence designed based on the aforementioned considerations: (i) two glycopeptides containing CS-E disaccharides at all faces of helix without (**1**) or with (**2**) backbone flexibility. (ii) One glycopeptide (**3**) containing a completely flexible backbone, representing a typical linear polymer scaffold. (iii) One glycopeptide (**4**) containing equally distributed CS-E disaccharides along the helix backbone but with increased spacing compared to **1**. (iv) One glycopeptide (**5**) displaying all CS-E disaccharides at only one face of the PPII helix. (v) Two control glycopeptides (**6** and **7**) containing unsulfated CS disaccharides. All CS-E glycopeptides differ subtly in their orientation of functional motifs yet contain an identical number of sugar units.

**Scheme 1:**
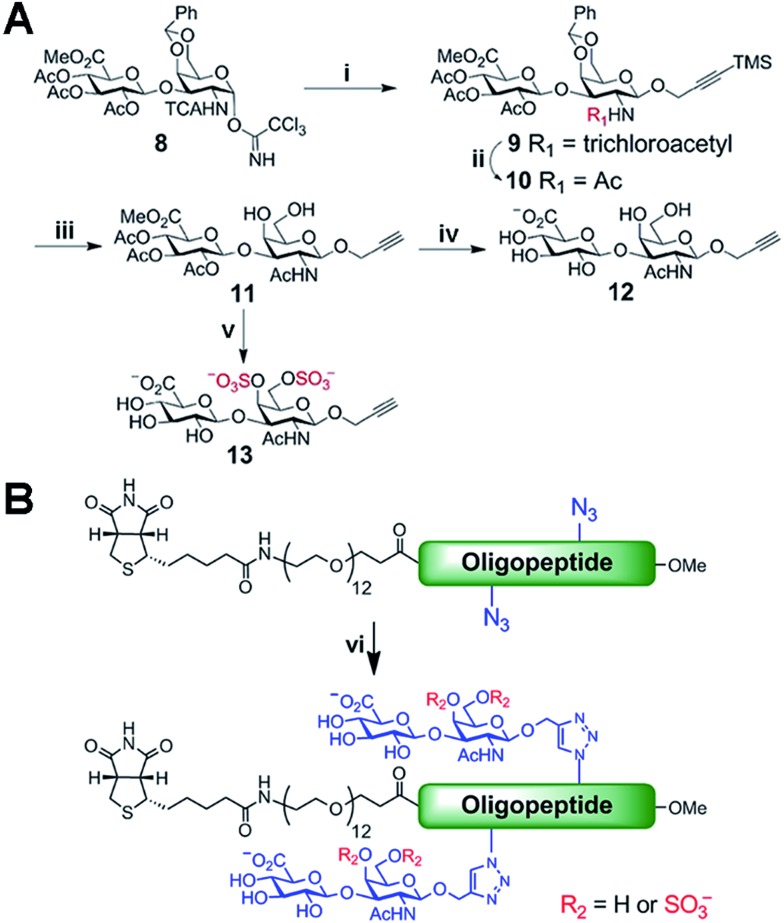
(A) Synthesis of alkyne-functionalized CS disaccharide monomers. (B) Click reaction for the synthesis of glycopeptide **1–7**. *Reagents and conditions*: (i) 3-TMS propargyl alcohol, TMSOTf, CH_2_Cl_2_, –20 °C, 61%; (ii) AIBN, Bu_3_SnH, toluene, 80 °C, 91%; (iii) AcOH–H_2_O (4 : 1), 80 °C; then, TBAF, 0 °C, 53% for 2 steps; (iv) LiOH, H_2_O_2_, THF–H_2_O, 0 °C to rt; then, NaOH, MeOH, rt, 95%; (v) SO_3_·TMA, DMF, 50 °C, 82%; then, step (iv), 91%; (vi) CuI, TBTA, DIPEA, DMSO, rt.

Biologically active CS disaccharides containing alkyne functionality were synthesized as described in Scheme 1. Briefly, the trichloroacetimidate **8** ^10^ was converted to the fully protected disaccharide **9** mediated by trimethylsilyl (TMS) triflate in good yield and stereoselectivity. Radical-mediated reduction of the *N*-trichloroacetyl group yielded the acetamide **10**. Notably, terminal alkyne was protected with TMS to avoid free-radical hydrostannation.^11^ Hydrolysis of the benzylidene acetal followed by removal of the TMS group afforded the diol **11**, which efficiently delivered sulfated disaccharide with SO_3_·trimethylamine complex. The desired CS-E disaccharide **13** was successfully elaborated by sequential treatment with LiOOH and NaOH. Deprotection of **11** under similar conditions furnished the unsulfated disaccharide **12** (Scheme 1A). As typical Fmoc chemistry on solid-phase resulted in very low coupling efficiency, all polyproline derivatives were prepared *via* standard Boc chemistry in the solution phase (see ESI† for synthetic details). Glycopeptides were prepared by conjugating CS disaccharides to azidopolyprolines *via* click reaction in the presence of copper(i) iodide (Scheme 1B). The disappearance of the azide vibrational band at 2100 cm^–1^ in the FT-IR spectra and the appearance of the characteristic peaks from both 1,2,3-triazole linkage and CS disaccharides in ^1^H NMR spectra of glycopeptides demonstrated the completion of the coupling reactions (Fig. S7 and NMR spectra in ESI†).^12^

With the glycopeptides successfully prepared, we performed circular dichroism (CD) studies to investigate their PPII helix stability. Although several reports have suggested that polyprolines can be efficiently functionalized while retaining PPII conformations,^9b–d^ we decided to confirm if the bulky and highly charged pendant sugars could induce a conformational change of backbones and thwart efforts to position functional groups at desired sites. For CD measurements, the peptide solutions (200 μM) were incubated at 4 °C for 24 h to allow for complete folding, and studied at room temperature. In order to examine exclusively the backbone conformation, we subtracted CD signals of corresponding CS disaccharides (Fig. S8B†)^13^ from those of glycopeptides. The CD traces demonstrated that all glycopeptides adopted PPII helical profiles with maximum positive bands at 224–228 nm and minimum negative bands at 208–213 nm, whereas **3** exhibited a random coil conformation (Fig. S8A†).^14^ These results indicate that the inclusion of CS disaccharides did not affect the PPII helical structure of our polyproline scaffolds, enabling us to create fine-tuned carbohydrate displays.

**Fig. 2:**
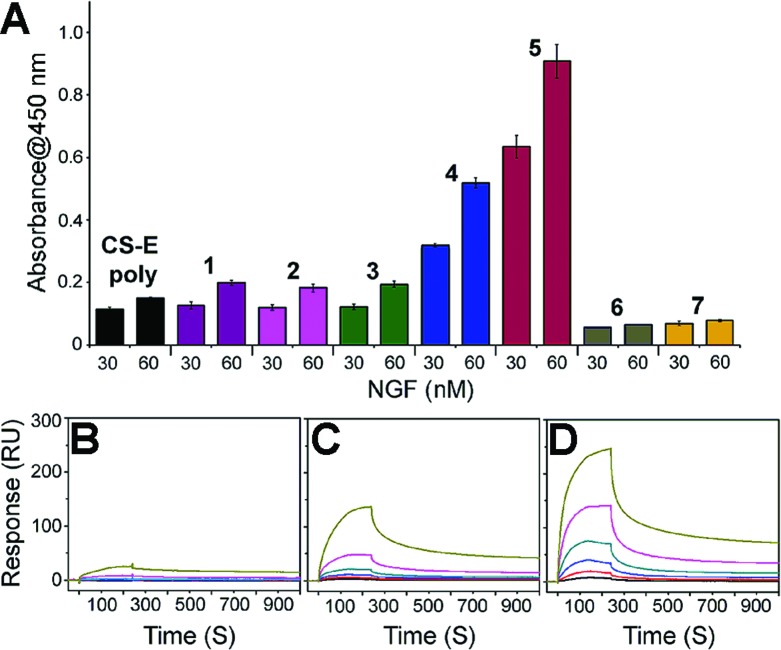
(A) ELISA graph depicting relative binding of NGF (30 and 60 nM concentration) to the indicated glycopeptides immobilized on streptavidin-coated 96-well plates. All data are presented as mean ± SD of triplicates. See Fig. S9† for the data with the full range of NGF concentrations. SPR sensorgrams for NGF binding at various concentrations (370, 185, 93, 46, 23, and 12 nM from top to bottom) to (B) CS-E polysaccharides, (C) **1**, and (D) **5**. See Table S3† for the complete kinetic parameters including equilibrium constants *K*_D_ with standard errors.

We next set about evaluating the ability of glycopeptides to tailor multivalent interactions with NGF using ELISA (Fig. 2A and S9†). Glycopeptides or CS-E polysaccharides were immobilized on plates as a mimic of GAG–extracellular protein interactions on the cell surface. NGF bound to both natural polysaccharides and **1** in a concentration-dependent manner, yet displayed no detectable binding to unsulfated **6**, confirming previous results that the CS-E sulfation motif provides a major binding epitope to NGF.^15^ To test whether the rigidity of polyproline would hinder protein binding, we evaluated the partially flexible **2** and entirely flexible **3**. However, no significant change in binding affinity was observed compared to **1**. We next investigated the influence of binding epitope density with **4**. In this design, proline residues were added between sugar conjugated prolines to allow for extra space while retaining the same carbohydrate configuration as **1**. This modification gave a significant increase in binding affinity of 2.6-fold compared to **1**. Lastly, we investigated two glycopeptides with all CS units at one face, namely **5** and **7**. It was found that **5** was the most effective binding ligand, with a 4.6-fold increase in binding affinity compared to **1**, while unsulfated **7** did not exhibit any noticeable binding. These results are quite striking in that small shifts in carbohydrate configuration across the polymer backbone can strongly alter their interactions with proteins.

SPR technology was employed to facilitate quantitative, real-time kinetic analysis of glycopeptide–NGF binding. Biotinylated glycopeptides and CS-E polysaccharides were immobilized on streptavidin-coated surface at normalized levels by molecular weights, and the interactions with NGF were investigated as a function of NGF concentration. Sensorgrams in Fig. 2 indicate that all glycopeptides and CS-E polysaccharides were efficiently recognized by NGF in a dose-dependent manner (Fig. 2B–D). In addition, the glycopeptide **5** gave higher overall responses than both **1** and natural polysaccharides, indicating that **5** recruits NGF more efficiently than the others, consistent with previously described ELISA results. Further kinetic analyses using a Langmuir 1 : 1 binding model (Table S3†) demonstrated that **5** (*K*_D_ = 0.146 μM) binds to NGF more strongly than **1** (*K*_D_ = 3.57 μM) by approximately 24-fold. Moreover, the kinetic result suggests that the higher binding affinity of **5** to NGF is mainly attributed to the relatively fast initial association rate (*k*_a_ (**5**) = 1.34(±0.13) × 10^4^ M^–1^ s^–1^) compared to **1** (*k*_a_ (**1**) = 3.46(±0.12) × 10^2^ M^–1^ s^–1^). Importantly, both glycopeptides recruit NGF more efficiently than natural CS-E polysaccharides (*K*_D_ = 6.21 μM) despite a lower number of disaccharide units per chain, highlighting the importance of pre-organized arrays of sugar units in protein binding affinity. Collectively, both SPR and ELISA studies shed light on the promise of polyproline scaffold to enable systematic exploration into binding epitope configuration in multivalent interactions.

After successfully demonstrating the efficacy of our strategy in manipulating protein binding affinity, we sought to further validate whether our approach could be applied to modulate signal transduction in biological milieu. Signal transduction in certain neuronal differentiation processes requires association of NGF with the ectodomain of neurotrophic tyrosine kinase receptor type 1 (TrkA) on the cell surface^16^ which can be further promoted by direct involvement of cell-surface CS-E polysaccharides.^15^ The biological importance of the NGF/TrkA signaling in neurite survival and outgrowth marks it as a promising therapeutic target for treating neurodegenerative diseases such as Alzheimer's disease.^15^ As such, to probe the therapeutic potential of our glycopeptides, we attempted to systematically investigate their interactions with the NGF/TrkA complex and evaluate the extent to which each design facilitates the stabilization of this signaling complex *via* computational methods.

As the basic residues in the protein are of major importance in the recognition of anionic glycopeptides *via* electrostatic interactions, we first examined the entire surface of the NGF/TrkA complex to identify regions containing these residues. Interestingly, unlike NGF, only two positively charged residues (Arg312/Arg342) on TrkA were available for glycopeptide binding. We hypothesized that the ability of our glycopeptides to stabilize the NGF/TrkA complex would rely on their interactions across NGF and TrkA, suggesting that at least one of the two basic residues on TrkA would need to participate in glycopeptide binding. Thus, we docked each glycopeptide to these residues on TrkA, followed by optimizing interactions with nearby basic residues on NGF, and then identified potential hydrogen-bonding partners at the predicted binding sites. The results are summarized in Table S4.†

**Fig. 3:**
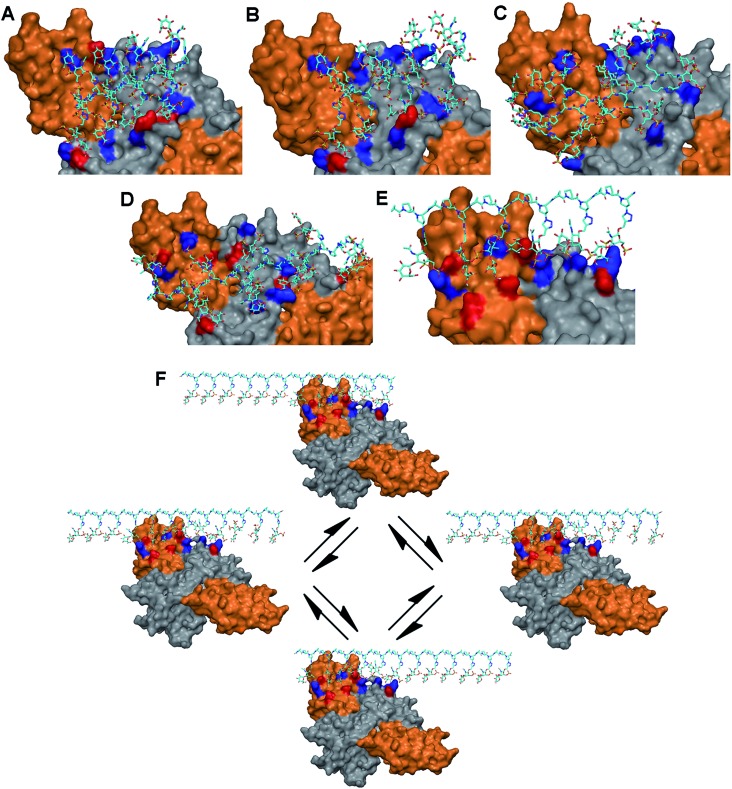
Proposed binding sites of (A) **1**, (B) **2**, (C) **3**, (D) **4** or (E) **5** for stabilization of the NGF (grey)/TrkA (orange) complex. Positively charged interacting residues are indicated in blue and potential hydrogen bonding partners in red. (F) Schematic illustration of statistical rebinding effect of **5** on the NGF/TrkA complex.

Our modeling studies demonstrated that each glycopeptide favors a distinct set of amino acid residues and presents unique electrostatic and hydrogen-bonding interactions to comprise the primary binding sites on the protein complex. **1** and **2** bind to two contiguous linear arrays of basic residues spaced approximately 25 Å apart on the protein complex: Lys34/Lys57/Arg69/Arg103 (NGF monomer: B) in one row, and Arg342 (TrkA)/Arg9 (NGF monomer A)/Lys74/Lys115 (NGF monomer: B) in the other, with the distance between the basic residues ranging from 10 to 14 Å in each array. The binding sites are further augmented by hydrogen-bonding partners for both glycopeptides (Fig. 3A and B, and Table S4†). On the other hand, although **4** shares multiple basic residues with **1** and **2** for complex binding due to the orientation of pendant sugars in three directions, the increased spacing between these sugars (∼20 Å) offers a better geometric fit to both basic residues on TrkA than **1** and **2**, implying an increased binding affinity to the TrkA component: Arg314/Arg342 (TrkA) for electrostatic interaction, and Ser312/Glu331 (TrkA) for hydrogen bonding (Fig. 3D). Similarly, glycopeptide **3** interacts with two contiguous arrays of several basic residues. However, the high degree of positional freedom of pendant sugars leads to the arrangement of these basic residues at irregular intervals ranging from 10 to 30 Å. Notably, no residue on the NGF/TrkA complex is predicted to engage in hydrogen bonding with **3** (Fig. 3C).

Unlike the other glycopeptides, **5** recognizes distinctively different arrays of basic residues on the protein complex. We found that its predicted binding site contains five contiguous basic residues arranged in a single linear array: Arg314/Arg342 (TrkA: monomer A), Arg9 (NGF: monomer A), and Lys74/Lys115 (NGF: monomer B). The distance between basic residues ranges from 10 to 17 Å, endowing them a good geometric fit to the pendant sugars located at one face of PPII helix. The binding site is further augmented by hydrogen-bonding partners in the region: Ser73 (NGF: monomer B) and Ser312/Glu324/Glu331/Glu334 (TrkA: monomer A) (Fig. 3E). Notably, molecular modeling suggests that **5** would possess higher binding affinity to TrkA than the other glycopeptides by displaying the greatest number of electrostatic and hydrogen-bonding interactions with TrkA. Additionally, we noted that only **5** would be favored by the statistical rebinding effect,^17^ known to be highly significant in multivalent interactions, due to the high local concentration of binding sugars. In this case, the dissociated binding elements on the protein complex from **5** can readily rebind to pendant sugars in close proximity as illustrated in Fig. 3F, leading to an increased binding affinity to both components of the NGF/TrkA complex. Taken together, our modeling study results suggest that **5** would be a promising candidate for enhancing NGF-mediated neuronal signaling.

**Fig. 4:**
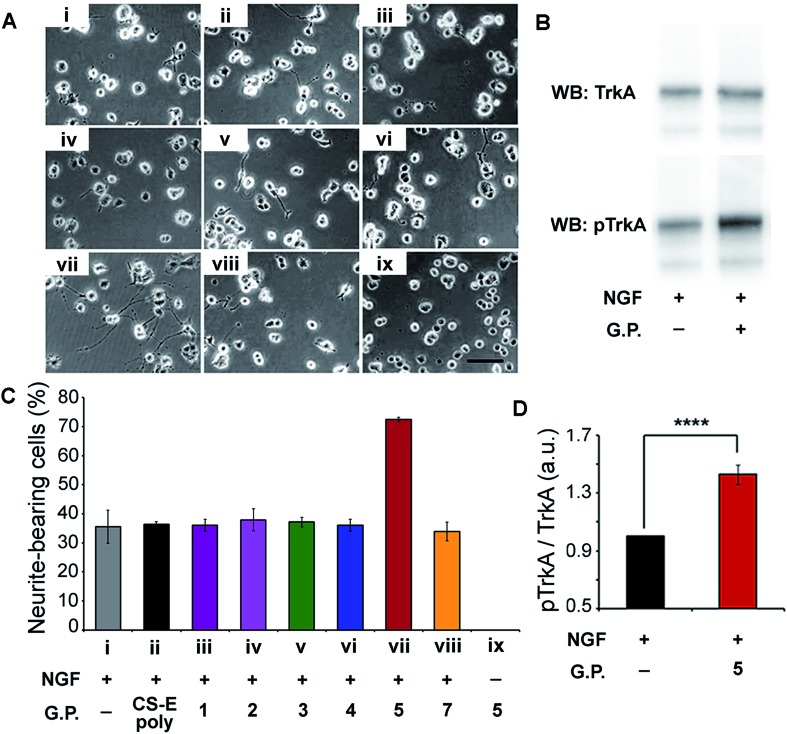
(A) Representative images of PC12 cells cultured on a substratum of laminin with the various glycopeptides. Conditions used are indicated in the *x*-axis section of (B). Fixed concentrations of 4 ng mL^–1^ for NGF, 10 μM for glycopeptides, and 2 μM for CS-E polysaccharides were used. (B) Western Blot analysis of PC12 cell lysates by blotting for total TrkA and phospho-TrkA (Tyr490). Serum-starved cells were exposed to NGF (4 ng mL^–1^) for 5 min with or without glycopeptide **5** (10 μM). (C) Statistical analysis of percentage of neurite-bearing cells. Error bars represent SD from three separate experiments. (D) Densitometric quantification of TrkA activation, calculated by dividing pTrkA signal by total TrkA signal and normalized with respect to the NGF-treated control. *n* = 5, ****p < 0.0001. See ESI† for details.

Verification of our molecular modeling predictions was done by measuring the ability of the different glycopeptides to modulate NGF-mediated neuronal differentiation of PC12 cells.^16b,18^ Cells were cultured on laminin-coated glass coverslips and treated with either glycopeptides or CS-E polysaccharides. Sulfated glycopeptide **5** exhibited strong neuritogenic activity in PC12 cells as predicted in our molecular model. Neurite extension was dramatically stimulated, and the percentage of primary neurite bearing cells was increased from 35% in the control to over 70% (Fig. 4A and C). Moreover, no neurites were observed for cells cultured in NGF-free medium even with **5**. Exogenous **5** was observed to raise the level of NGF-mediated TrkA activation by 43% relative to NGF control based on Western Blotting (Fig. 4B and D), further confirming that **5** functions through the NGF/TrkA pathway. In contrast, other sulfated glycopeptides (**1**, **2** and **4**), and unsulfated **7** (a control for **5**) showed only little or no effect. It is noteworthy that random coil **3**, sharing structural similarity to a typical linear polymer scaffold, also did not exhibit enhanced neuritogenic activity, highlighting the significance of the defined spatial orientation of **5**. We note that the lack of an expected inhibition effect of exogenously introduced natural CS-E polysaccharides is caused by the laminin substratum neutralizing the inhibitory activity of CS.^19^ These cellular results further support our hypothesis that the accurate spatial orientation of sulfated sugars on target protein surface is vital for high specificity in protein recognition, and confirm our previous molecular modeling predictions of **5** as the best enhancer of NGF/TrkA signaling. The enhanced neuritogenesis of cells co-treated with NGF and **5** strongly suggest that **5** may find therapeutic application as an agent or adjuvant in the treatment of neurodegenerative disease.

## Conclusions

In conclusion, we have engineered a new class of CS glycopeptides in which the display of pendant disaccharides has been precisely controlled using a polyproline scaffold. Our protein binding and cellular studies have demonstrated the efficacy of our design to fine-tune protein recognition events and thereby accurately tailor specific biological pathways. Importantly, we have successfully applied our approach to identify an effective neuronal promoting agent for selectively modulating the NGF-mediated signaling pathway, offering a potential therapeutic strategy for the treatment of neurodegenerative disease. We anticipate that our findings will open up the prospect of developing highly specific therapeutic methods associated with GAG-mediated events.
