# Supplementary material for: Tailored chondroitin sulfate glycomimetics via a tunable multivalent scaffold for potentiating NGF/TrkA-induced neurogenesis
Source: Chem Sci. 2014 Oct 15;6(1):450–6. doi: 10.1039/c4sc02553a (PMC5485393; doi:10.1039/c4sc02553a)
Supplement: Supplementary file 1 [file SC-006-C4SC02553A-s001.pdf]

Electronic Supplementary Material (ESI) for Chemical Science.
This journal is © The Royal Society of Chemistry 2014

-Supplementary Information-

Tailored Chondroitin Sulfate Glycomimetics via a Tunable Multivalent Scaffold for Potentiating NGF/TrkA-Induced Neurogenesis

Pei Liu,‡ Liwei Chen,‡ Jerry K. C. Toh, Yi Li Ang, Joo-Eun Jee, Jaehong Lim, Su Seong Lee, and Song-Gil Lee*

Institute of Bioengineering and Nanotechnology, 31 Biopolis Way, The Nanos, Singapore 138669

General Methods

Unless otherwise stated, reactions were performed in flame-dried glassware under argon atmosphere and using anhydrous solvents. All commercially obtained reagents were used as received unless otherwise noted. Thin layer chromatography (TLC) was performed using E. Merck silica gel 60 F254 precoated plates (0.25 mm). Visualization of the developed chromatogram was performed by UV, cerium ammonium molybdate or ninhydrin stain as necessary. Merck silica gel 60 (particle size 0.040 - 0.063 mm) was used for flash chromatography. Gel filtration chromatography (Sephadex G-15 ultrafine) was used in order to achieve purification of the glycopeptides.

^{1}H NMR and proton decoupling experiments were recorded on a Bruker AVIII 400 (400 MHz) spectrometer and are reported in parts per million (δ) relative to $CDCl_3$ (7.26 ppm), CD_3OD (4.87 ppm), and D_2O (4.80 ppm). Data for the ^{1}H NMR spectra are reported as follows: chemical shift (δ ppm), multiplicity (s = singlet, bs = broad singlet, d = doublet, t = triplet, q = quartet, m = multiplet), coupling constant in Hz, and integration. ^{13}C NMR spectra were obtained on a Bruker AVIII 400 (100 MHz) spectrometer and are reported in terms of chemical shift. Mass spectra were obtained from Chemical, Molecular and Materials Analysis Centre at the National University of Singapore.

3-Trimethylsilylpropargyl O-(methyl 2,3,4-tri-O-acetyl-β-D-glucopyranosyluronate)-(1→3)-4,6-O-benzylidene-2-deoxy-2-trichloroacetamido-β-D-galactopyranoside 9: Donor **8** (0.500 g, 0.573 mmol) was co-evaporated with toluene (3 x 5 mL) and dried under vacuum overnight. To a solution of **8** and 3-trimethylsilylpropargyl alcohol (0.425 mL, 2.863 mol) in dry CH_2Cl_2 (7.35 mL) was added 4Å powdered molecular sieves. The reaction was stirred at rt for 30 min, cooled to -78 °C, and then stirred for an additional 30 min. Trimethylsilyl trifluoromethanesulfonate (0.227 mM in CH_2Cl_2, 0.115 mmol, 500 μL) at -78 °C was added to the reaction dropwise. The reaction was warmed to -15 °C, stirred for 2 hrs, and quenched with triethylamine. The

reaction mixture was filtered through Celite and concentrated to afford yellow syrup. The product was purified by flash chromatography (1% → 2% 2-propanol:CH_2Cl_2) to afford slightly impure **9** as a light yellow solid. The impure **9** was then purified one more time by flash chromatography (2% → 5% THF:[1:2 hexanes:CH_2Cl_2]) to afford pure **9** (0.295 g, 61%) as a white solid. R_f = 0.45 (6:3:1 CH_2Cl_2:hexanes:THF). ^{1}H NMR (400 MHz, $CDCl_3$): δ 7.53 (m, 2 H, Ar*H*), 7.38 – 7.33 (m, 3 H, Ar*H*), 6.97 (d, *J* = 6.8 Hz, 1 H, N*H*), 5.59 (s, 1 H, PhC*H*), 5.23 (d, *J* = 8.2 Hz, 1 H, H-1'), 5.23 (t, *J* = 9.4 Hz, 1 H, H-4), 5.16 (t, *J* = 8.8 Hz, 1 H, H-3), 5.04 (t, *J* = 7.8 Hz, 1 H, H-2), 4.90 (d, *J* = 7.5 Hz, 1 H, H-1), 4.77 (dd, *J* = 11.1, 3.4 Hz, 1 H, H-3'), 4.46 (d, *J* = 3.3 Hz, 1 H, H-4'), 4.40 (s, 2 H, CH_2–C≡C), 4.33 (d, *J* = 12.4 Hz, 1 H, H-6'), 4.10 (d, *J* = 11.6 Hz, 1 H, H-6'), 4.03 (d, *J* = 9.7 Hz, 1 H, H-5), 3.83 – 3.77 (m, 1 H, H-2'), 3.72 (s, 3 H, OCH_3), 3.55 (s, 1 H, H-5'), 2.01 (s, 3 H, C(O)CH_3), 2.00 (s, 3 H, C(O)CH_3), 1.99 (s, 3 H, C(O)CH_3), 0.16 (s, 9 H, Si(CH_3)$_3$); ^{13}C NMR (100 MHz, $CDCl_3$): δ 170.20, 169.57, 169.32, 167.32, 162.27, 137.80, 128.99, 128.24, 126.33, 100.83, 100.40, 100.17, 96.10, 92.54, 92.48, 75.76, 74.22, 72.57, 72.12, 71.40, 69.22, 69.14, 66.76, 56.53, 55.03, 53.04, 20.97, 20.72, 20.63, -0.06; ESI MS: *m/z* calcd for [$C_{34}H_{42}Cl_3NO_{15}Si$ + Na]$^+$: 860.1287, obsd 860.1296.

3-Trimethylsilylpropargyl O-(methyl 2,3,4-tri-O-acetyl-β-D-glucopyranosyluronate)-(1→3)-4,6-O-benzylidene-2-deoxy-2-acetamido-β-D-galactopyranoside 10: Reduction of the trichloroacetamide group was performed using a procedure modified from Bélot *et. al.*[1] Disaccharide **9** (0.353 g, 0.421 mmol) was dissolved in toluene (8.7 mL), and tributylstannane (1132 μL, 4.21 mmol) and 2,2'- azobisisobutyronitrile (34.5 mg, 0.211 mmol) were added. After stirring at rt for 30 min, the reaction mixture was heated to 80 °C and stirred for an additional 4 h 30 min. The reaction was then cooled to rt and concentrated to afford a white solid. The product was purified by flash chromatography (3 → 11% THF:CH_2Cl_2) to afford the desired acetamide (0.281 g, 91%) as a white solid. R_f = 0.25 (4% THF:CH_2Cl_2). ^{1}H NMR (400 MHz, $CDCl_3$): δ 7.53 (m, 2 H, Ar*H*), 7.37 – 7.32 (m, 3 H, Ar*H*), 5.81 (d, *J* = 6.6 Hz, 1 H, N*H*), 5.56 (s, 1 H, PhC*H*), 5.25 – 5.18 (m, 3 H, H-3, H-4, H-1'), 5.01 (t, *J* = 7.7 Hz, 1 H, H-2), 4.92 – 4.88 (m, 2 H, H-1, H-3'), 4.43 – 4.36 (m, 3 H, H-4', CH_2–C≡C), 4.30 (d, *J* = 12.5 Hz, 1 H, H-6'), 4.08 – 4.02 (m, 2 H, H-5, H-6'), 3.70 (s, 3 H, OCH_3), 3.53 (s, 1 H, H-5'), 3.47 – 3.41 (m, 1 H, H-2'), 2.02 (s, 6 H, NHC(O)CH_3, C(O)CH_3), 2.00 (s, 3 H, C(O)CH_3), 1.98 (s, 3 H, C(O)CH_3), 0.18 (s, 9 H, Si(CH_3)$_3$); ^{13}C NMR (100 MHz, $CDCl_3$): δ 171.31, 170.29, 169.59, 169.18, 167.47, 137.90, 128.92, 126.37, 100.85, 100.81, 100.57, 97.07,

91.88, 75.98, 75.16, 72.52, 72.03, 71.74, 69.29, 69.18, 66.60, 56.75, 54.31, 52.98, 23.98, 20.86, 20.75, 20.65, -0.07; ESI MS: *m/z* calcd for $[C_{34}H_{45}NO_{15}Si + Na]^+$: 758.2456, obsd 758.2469.

2-Propargyl O-(methyl 2,3,4-tri-O-acetyl-β-D-glucopyranosyluronate)-(1→3)-2-deoxy-2-acetamido-β-D-galactopyranoside 11: The acetamide (0.353 g, 0.480 mmol) was dissolved in AcOH/water (4:1, 3.0 mL) and stirred at 80 °C. After 30 min, the reaction mixture was cooled and concentrated. The resulting concentrate was co-evaporated with toluene (3 x 3 mL) for the complete removal of AcOH.

To a solution of crude diol (0.242 g, 0.374 mmol) in THF (3.7 mL) was added TBAF (1 M solution in THF, 0.448 mmol, 448 μL) and the mixture stirred at 0 °C for 1.5 hr. At this time the addition of Amberlyst IR-120 resin was made and the reaction was stirred for a further 30 min. After filtration, the mixture was concentrated to afford a light yellow solid. The residue was purified via flash chromatography (5 → 7% MeOH:CH_2Cl_2) to afford the desired compound (0.147 g, 53%) as a white solid. R_f = 0.30 (10% MeOH:CH_2Cl_2). ^1H NMR (400 MHz, CD_3OD): δ 5.35 (t, *J* = 6.6 Hz, 1 H, H-3), 5.11 (t, *J* = 9.8 Hz, 1 H, H-4), 5.00 (dd, *J* = 8.0 Hz, 9.4 Hz, 1 H, H-2), 4.91 (d, *J* = 8.0 Hz, 1 H, H-1), 4.66 (d, *J* = 8.2 Hz, 1 H, H-1'), 4.43 – 4.37 (m, 2 H, CH_2–C≡C), 4.29 (d, *J* = 10.0 Hz, 1 H, H-5), 4.07 (bs, 1 H, H-4'), 4.00 – 3.98 (m, 1 H, H-2'), 3.91 – 3.89 (m, 1 H, H-3'), 3.81 – 3.72 (m, 2 H, H-6'), 3.75 (s, 3 H, OCH_3), 3.54 (t, *J* = 5.8 Hz, 1 H, H-5'), 2.87 (t, *J* = 2.32 Hz, 1 H, C≡C*H*), 2.06 (s, 3 H, NHC(O)CH_3), 2.03 (s, 3 H, C(O)CH_3), 2.01 (s, 3 H, C(O)CH_3), 2.00 (s, 3 H, C(O)CH_3); ^{13}C NMR (100 MHz, CD_3OD): δ 173.41, 171.48, 171.21, 169.31, 102.60, 100.50, 81.36, 80.14, 76.47, 76.13, 73.45, 72.95, 72.57, 70.84, 69.17, 62.40, 56.43, 53.41, 52.56, 23.40, 20.97, 20.77, 20.49, 20.41; ESI MS: *m/z* calcd for $[C_{24}H_{33}NO_{15} + Na]^+$: 598.1748, obsd 598.1760.

2-Propargyl O-(sodium-β-D-glucopyranosyluronate)-(1→3)-2-deoxy-2-acetamido-*β*-D-galactopyranoside 12: Compound **11** (40 mg, 0.0695 mmol) was dissolved in THF (684 μL) and H_2O (388 μL) and cooled to 0 °C. To this were added 1 M aq. LiOH (270 μL) and 30% H_2O_2 (135 μL). The reaction stirred at 0 °C for 1 hr and at rt for 12 hr. At this time, 4 M NaOH (203 μL) and MeOH (1008 μL) were added and the reaction stirred for another 12 hr.[2] It was neutralized with Amberlyst IR-120 resin, filtered, and lyophilized to afford an orange solid. The product was purified by Sephadex G-15 (100% H_2O) and lyophilized to afford **12** as a white solid (28.7 mg, 95%). ^1H NMR (400 MHz, D_2O): δ 4.70 (d, *J* = 8.6 Hz, 1 H, H-1'), 4.52 (d, *J* = 7.8 Hz, 1 H, H-1), 4.44 – 4.42 (m, 2 H, CH_2–C≡C), 4.18 (d, *J* = 3.1 Hz, 1 H, H-4'), 4.02 (dd, *J* = 10.9, 9.8 Hz, 1 H, H-2'), 3.86 (dd, *J* = 10.8, 3.2 Hz, 1 H, H-3'), 3.81 – 3.69 (m, 4 H, H-5, H-5', H-6', H-6'), 3.53 – 3.46 (m, 2 H, H-3, H-4), 3.37 – 3.32 (m, 1 H, H-2), 2.91 (t, *J* = 2.4 Hz,

1 H, C≡C*H*), 1.94 (s, 3 H, NHC(O)CH_3); ^{13}C NMR (100 MHz, D_2O): δ 175.38, 174.93, 104.10, 99.57, 80.11, 78.84, 78.83, 76.07, 75.85, 75.25, 75.01, 72.64, 71.68, 67.67, 60.98, 56.56, 50.94, 22.25; ESI MS: *m/z* calcd for $[C_{17}H_{25}NO_{12} - H]^-$: 434.1299, obsd 434.1308.

2-Propargyl O-(methyl 2,3,4-tri-O-acetyl-β-D-glucopyranosyluronate)-(1→3)-4,6-di-*O*-sodium sulfonato-2-deoxy-2-acetamido-*β*-D-galactopyranoside: To a solution of diol **11** (0.050 g, 0.087 mmol) in DMF (3.9 mL) was added sulfur trioxide trimethylamine complex (SO_3•TMA) (0.305 g, 2.17 mmol). The reaction mixture was stirred at 50 °C overnight and then cooled to rt. The product was purified on Sephadex LH-20 (50% MeOH:CH_2Cl_2), followed by silica gel chromatography (5% → 20% MeOH:CH_2Cl_2) to afford a sulfated disaccharide (0.053 g, 82%) as a white solid. R_f = 0.20 (20% MeOH:CH_2Cl_2). ^{1}H NMR (400 MHz, D_2O): δ 5.43 – 5.39 (m, 1 H, H-3), 5.27 (t, *J* = 9.9 Hz, 1 H, H-4), 5.02 – 5.00 (m, 2 H, H-1, H-2), 4.89 (bs, 1 H, H-4'), 4.78 (d, *J* = 8.6 Hz, 1 H, H-1'), 4.46 (dd, *J* = 4.0, 2.4 Hz, 2 H, CH_2−C≡C), 4.42 (d, *J* = 9.9 Hz, 1 H, H-5), 4.32 (dd, *J* = 11.5, 3.4 Hz, 1 H, H-6'), 4.26 – 4.21 (m, 1 H, H-6'), 4.11 – 4.01 (m, 3 H, H-2', H-3', H-5'), 3.80 (s, 3 H, OCH_3), 2.85 (bs, 1 H, C≡C*H*), 2.11 (s, 3 H, NHC(O)CH_3), 2.10 (s, 6 H, C(O)CH_3, C(O)CH_3), 2.07 (s, 3 H, C(O)CH_3); ^{13}C NMR (100 MHz, D_2O): δ 174.45, 173.01, 172.70, 172.43, 169.34, 100.59, 100.00, 99.27, 78.72, 78.70, 76.34, 76.29, 75.73, 72.52, 72.39, 71.44, 71.32, 69.43, 67.67, 56.91, 53.50, 51.09, 22.23, 20.15, 20.00, 19.92; ESI MS: *m/z* calcd for $[C_{24}H_{33}NO_{21}S_2 - H]^-$: 734.0908, obsd 734.0903.

2-Propargyl O-(sodium-β-D-glucopyranosyluronate)-(1→3)-4,6-di-*O*-sodium sulfonato-2-deoxy-2-acetamido-*β*-D-galactopyranoside 13: The sulfated compound (185 mg, 0.251 mmol) was dissolved in THF (684 μL) and H_2O (338 μL) and cooled to 0 °C. To this were added 1 M aq. LiOH (270 μL) and 30% H_2O_2 (135 μL). The reaction was stirred at 0 °C for 1 hr and at rt for 12 hr. At this time, 4 M aq. NaOH (203 μL) and MeOH (1008 μL) were added and the reaction was stirred for another 12 hr. It was then neutralized with Amberlyst IR-120 resin, filtered, and lyophilized to afford an orange solid. The product was purified by Sephadex G-15 (100% H_2O) and lyophilized to afford **13** as a white solid (136 mg, 91%). ^{1}H NMR (400 MHz, D_2O): δ 4.86 (d, *J* = 2.4 Hz, 1 H, H-4'), 4.78 (d, *J* = 9.0 Hz, 1 H, H-1'), 4.70 (d, *J* = 7.8 Hz, 1 H, H-1), 4.45 (dd, *J* = 4.1, 2.4 Hz, 2 H, CH_2−C≡C), 4.31 (dd, *J* = 11.4, 3.3 Hz, 1 H, H-6'), 4.24 – 4.19 (m, 1 H, H-6'), 4.13 – 4.04 (m, 3 H, H-2', H-3', H-5'), 3.90 (d, *J* = 9.8 Hz, 1 H, H-5), 3.61 (t, *J* = 9.1 Hz, 1 H, H-4), 3.50 (t, *J* = 9.3 Hz, 1 H, H-3), 3.40 (dd, *J* = 9.4, *J* = 7.8 Hz, 1 H, H-2), 2.92 (t, *J* = 2.4 Hz, 1 H, C≡C*H*), 2.04 (s, 3 H, NHC(O)CH_3); ^{13}C NMR (100 MHz, D_2O): δ 177.46, 175.82, 106.10,

101.93, 81.26, 78.75, 78.60, 78.07, 77.57, 77.44, 74.95, 74.83, 73.81, 70.30, 59.45, 53.93, 24.81; ESI MS: *m/z* calcd for $[C_{17}H_{25}NO_{18}S_2 - H]^-$: 594.0435, obsd 594.0429.

General Procedure for Solution Phase Peptide Synthesis: In a typical solution phase peptide synthesis, Boc-Peptide(1)-OH (1.0 equiv.) was dissolved in DMF (final concentration was adjusted to 0.1M). H-Peptide(2)-OCH_3 (1.1 equiv.), TBTU (1.1 equiv.), and DIPEA (5.0 equiv.) were then added to the solution. The reaction mixture was stirred at room temperature until the reaction was completed. After the completion, the solvent was removed *in vacuo* to afford a yellow solid. Purification of this solid by either flash column chromatography on silica or reverse phase HPLC afforded Boc-Peptide(1)-Peptide(2)-OCH_3.

General method for Boc group deprotection of Boc-Peptide(1)-Peptide(2)-OCH_3: Boc-Peptide(1)-Peptide(2)-OCH_3 was dissolved in CH_2Cl_2/TFA (1:1) at 0 °C, and stirred for 2 hrs. After the completion, the solvent was removed by blowing a slow stream of nitrogen gas and then diethyl ether was added to give H-Peptide(1)-Peptide(2)-OCH_3 quantitatively as a white precipitate.

General procedure for methyl group deprotection of Boc-Peptide(1)-Peptide(2)-OCH_3: To a solution of Boc-Peptide(1)-Peptide(2)-OCH_3 (1.0 equiv.) in THF/MeOH (1:1, final concentration was adjusted to 0.05M) was added aq. NaOH (2.0 equiv.) and sonicated for 5 min. At this time, the same volume of CH_2Cl_2 was added and the reaction was stirred for additional 2 hrs. It was then neutralized with Amberlyst IR-120 resin, filtered, and evaporated *in vacuo* to afford Boc-Peptide(1)-Peptide(2)-OH quantitatively.

General procedure for the synthesis of Biotin-PEG12-Azidopolyproline-OCH_3: To a solution of H-Azidopolyproline-OCH_3 (1.0 equiv.) in DMSO (final concentration was adjusted to 0.1M) were added EZ-link NHS-PEG12-Biotin (1.2 equiv., from Thermo Scientific) and DIPEA (5.0 equiv.), and stirred overnight at room temperature. After the completion, the solvent was removed *in vacuo* to afford a yellow sticky solid. Purification of this solid by reverse phase HPLC afforded biotin-PEG12-azidopolyproline-OCH_3.

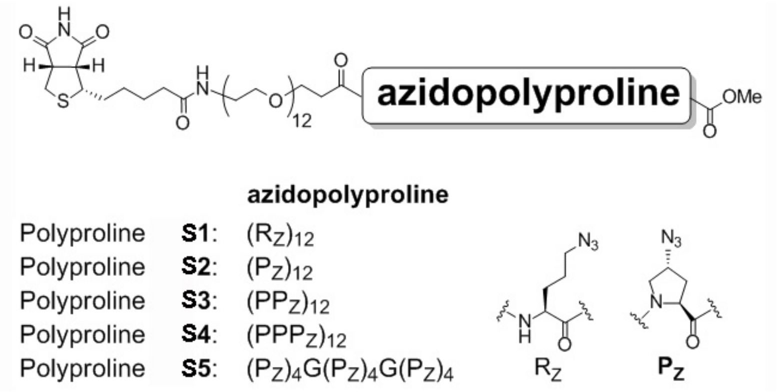

Figure S1. Structures of biotin-PEG12-azidopolyproline-OCH_3.

Table S1. Analytical HPLC conditions for biotin-PEG12-azidopolyproline-OCH_3.

Biotin-PEG12-azidopolyproline-OCH_3	HPLC condition *	Retention time
Polyproline S**1**	5 – 60% A over 8 min	7.35 min
Polyproline S**2**	5 – 60% A over 8 min	5.30 min
Polyproline S**3**	5 – 60% A over 8 min	4.27 min
Polyproline S**4**	5 – 60% A over 8 min	2.40 min
Polyproline S**5**	5 – 60% A over 8 min	4.93 min

* A: CH_3CN/0.1%TFA, B: H_2O/0.1%TFA, %A + %B = 100%.

Table S2. ESI-MS Data of biotin-PEG12-azidopolyproline-OCH_3.

Biotin-PEG12-azidopolyproline-OCH_3	MS, calculated	ESI-MS, observed
Polyproline S**1**	2538.29	1270.94 ($[M+2H]^{2+}$), 847.62 ($[M+3H]^{3+}$)
Polyproline S**2**	2514.11	1258.87 ($[M+2H]^{2+}$), 839.39 ($[M+3H]^{3+}$)
Polyproline S**3**	3678.74	1841.03 ($[M+2H]^{2+}$), 1227.79 ($[M+3H]^{3+}$), 921.35 ($[M+4H]^{4+}$)
Polyproline S**4**	4843.37	1616.56 ($[M+3H]^{3+}$), 1212.66 ($[M+4H]^{4+}$)
Polyproline S**5**	2628.15	1315.81 ($[M+2H]^{2+}$), 877.92 ($[M+3H]^{3+}$)

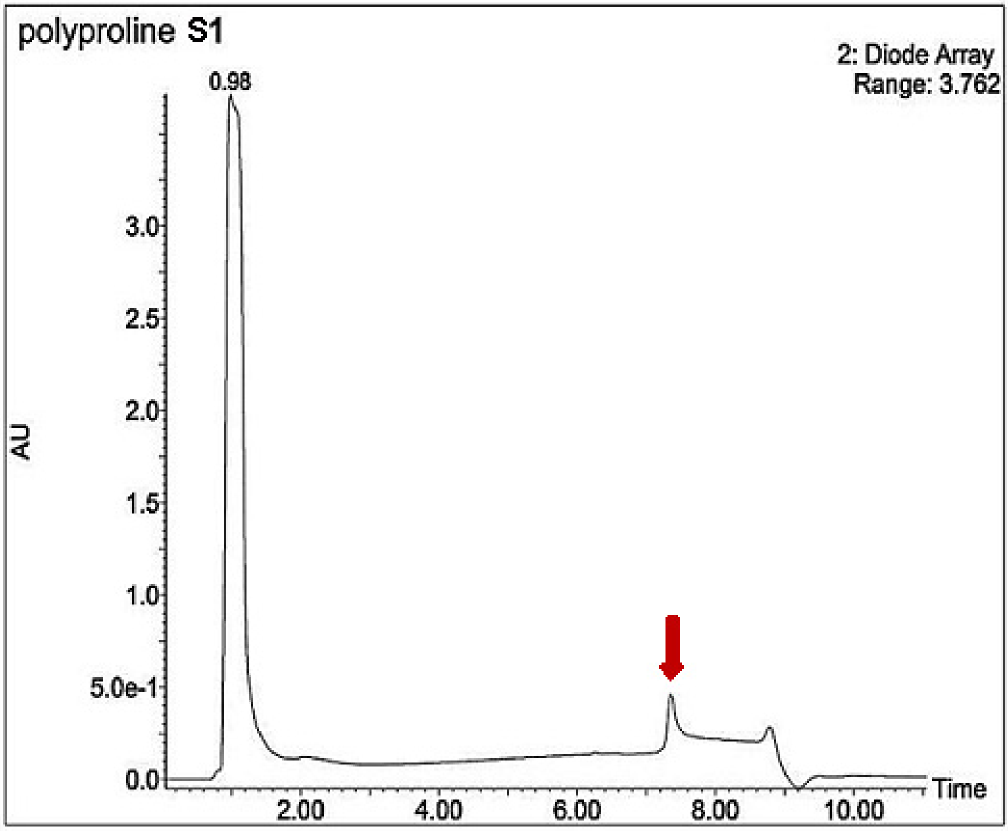

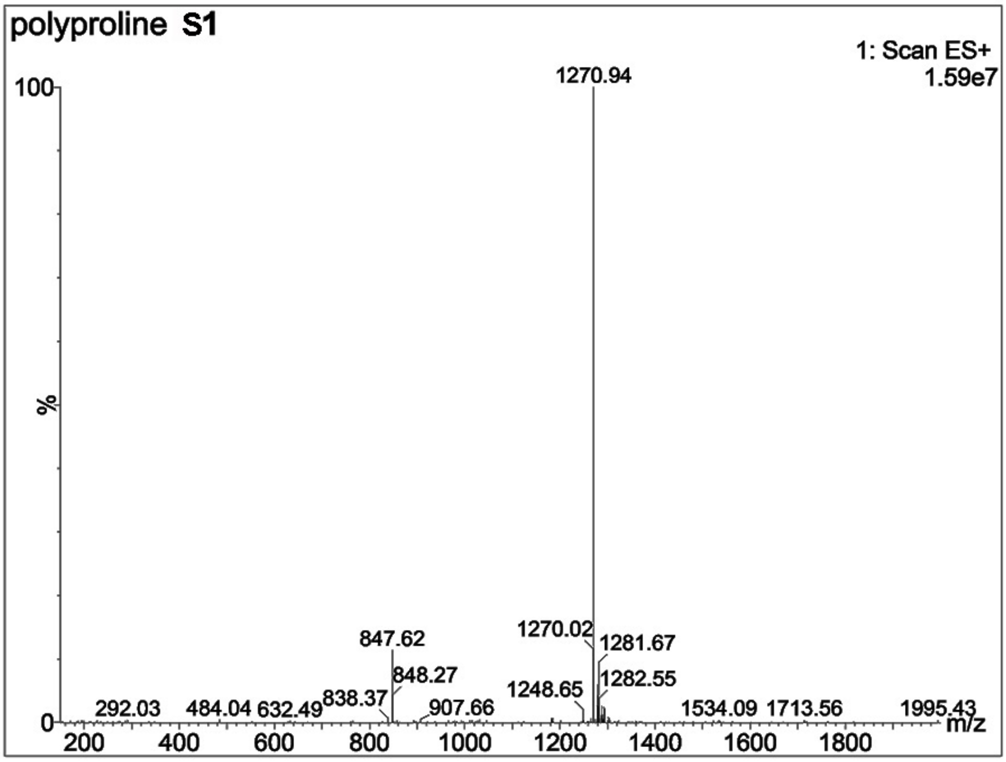

Figure S2. Analytical HPLC traces (top) and ESI mass data (bottom) of polyproline S**1**.

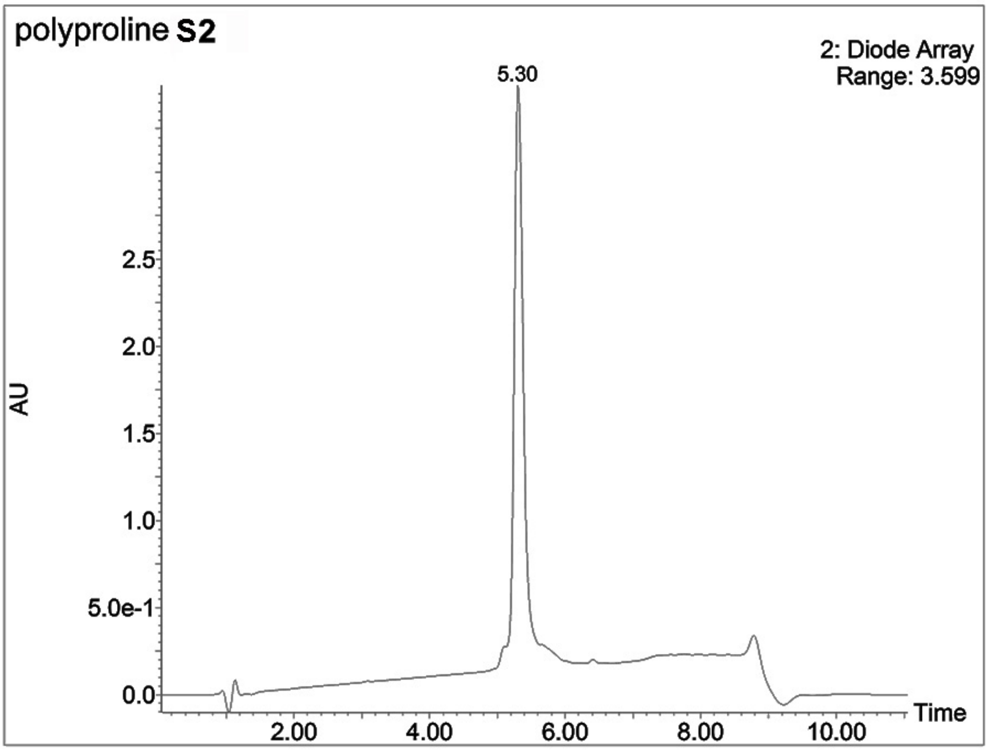

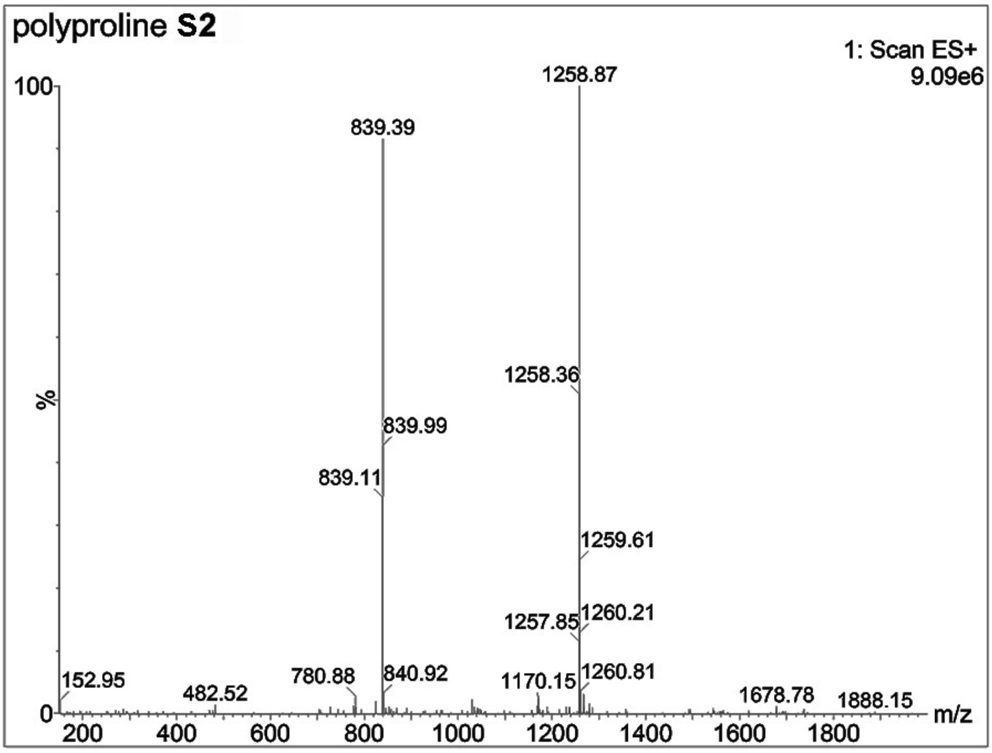

Figure S3. Analytical HPLC traces (top) and ESI mass data (bottom) of polyproline S**2**.

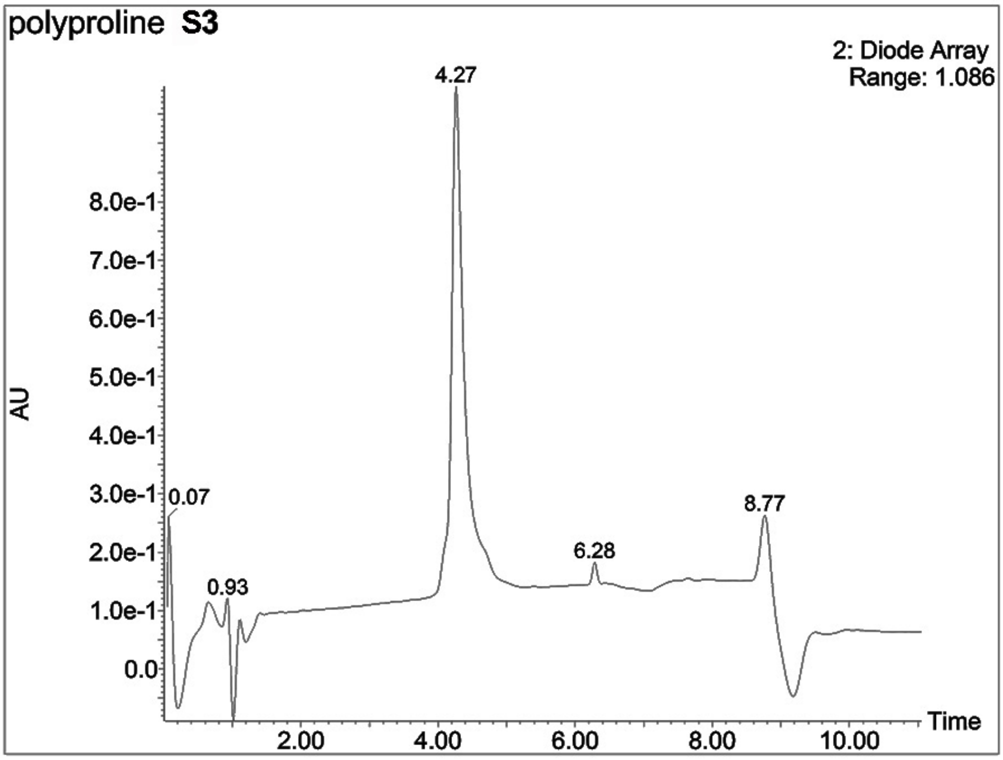

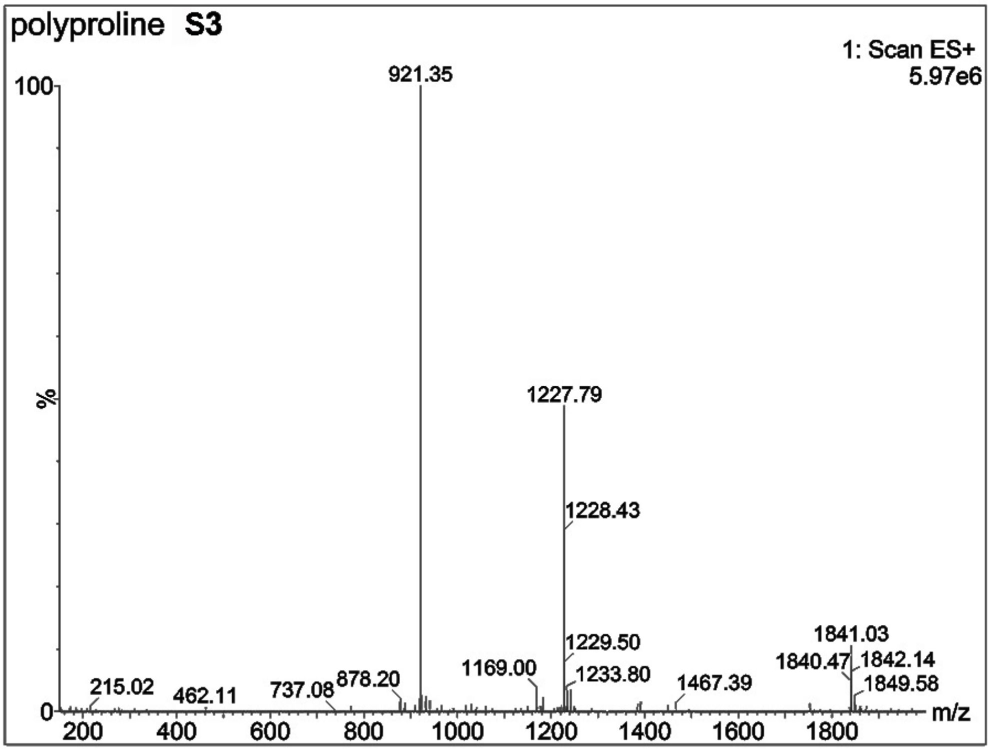

Figure S4. Analytical HPLC traces (top) and ESI mass data (bottom) of polyproline S**3**.

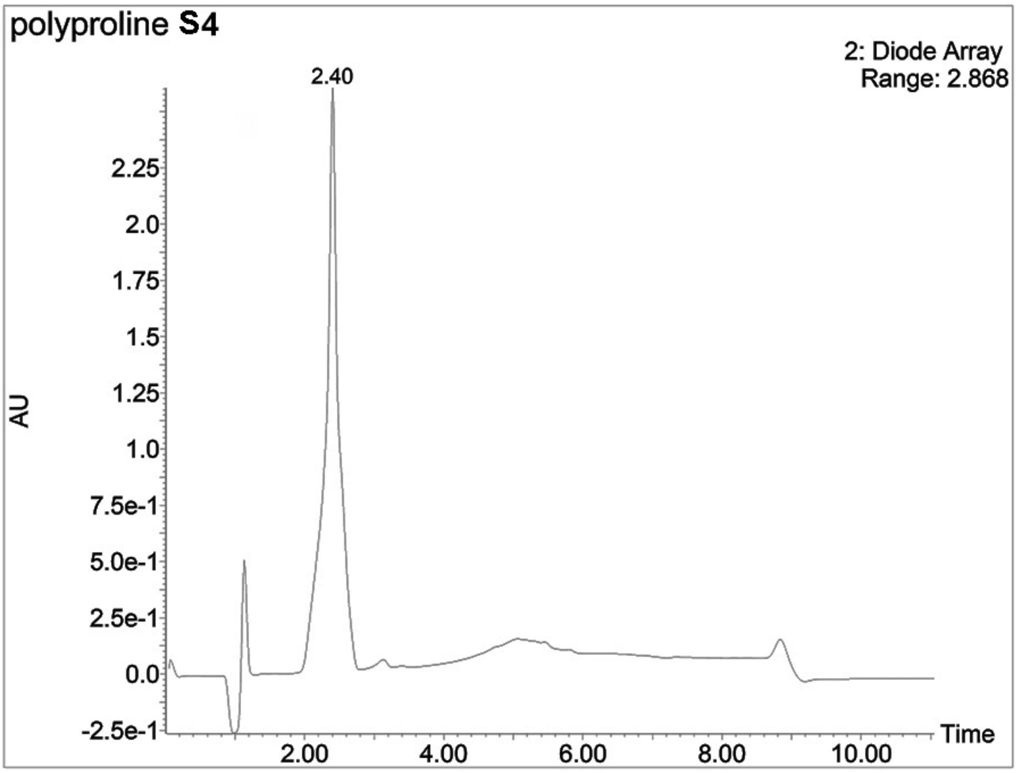

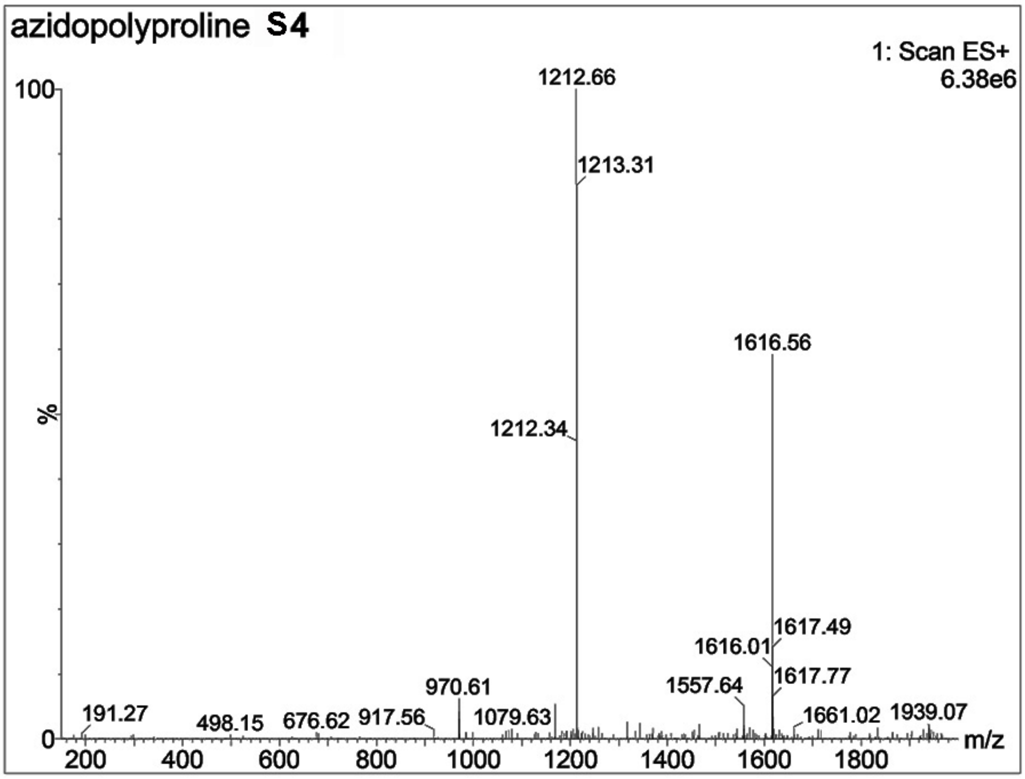

Figure S5. Analytical HPLC traces (top) and ESI mass data (bottom) of polyproline S**4**.

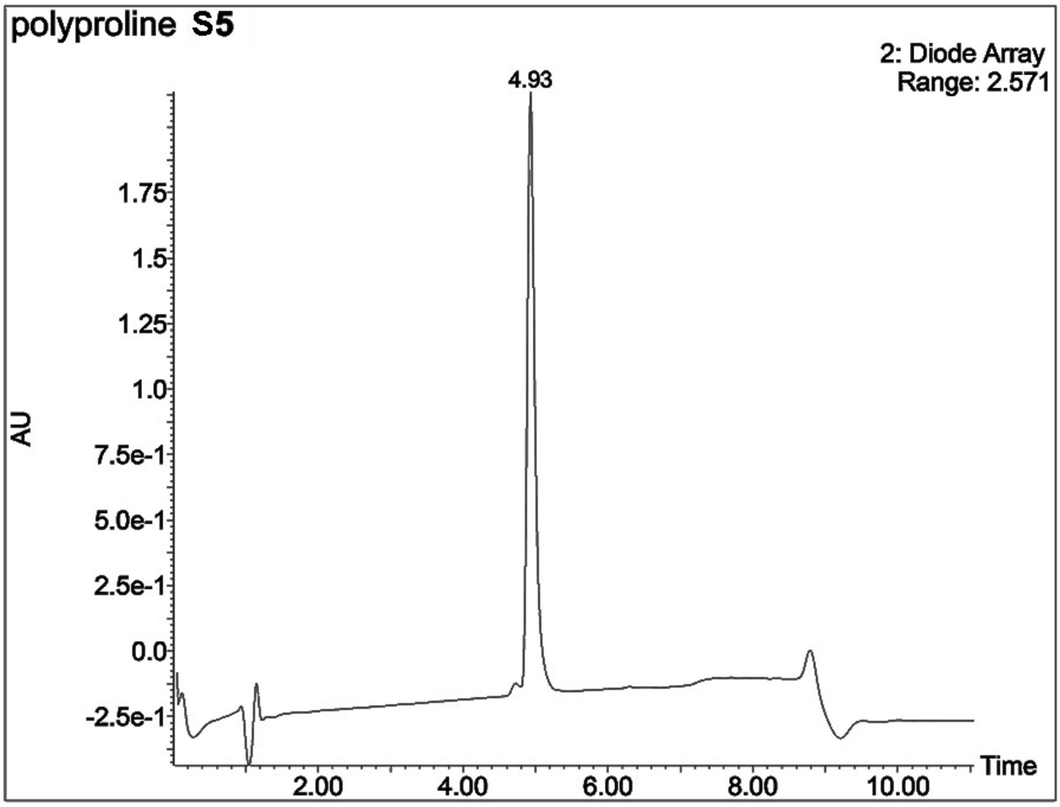

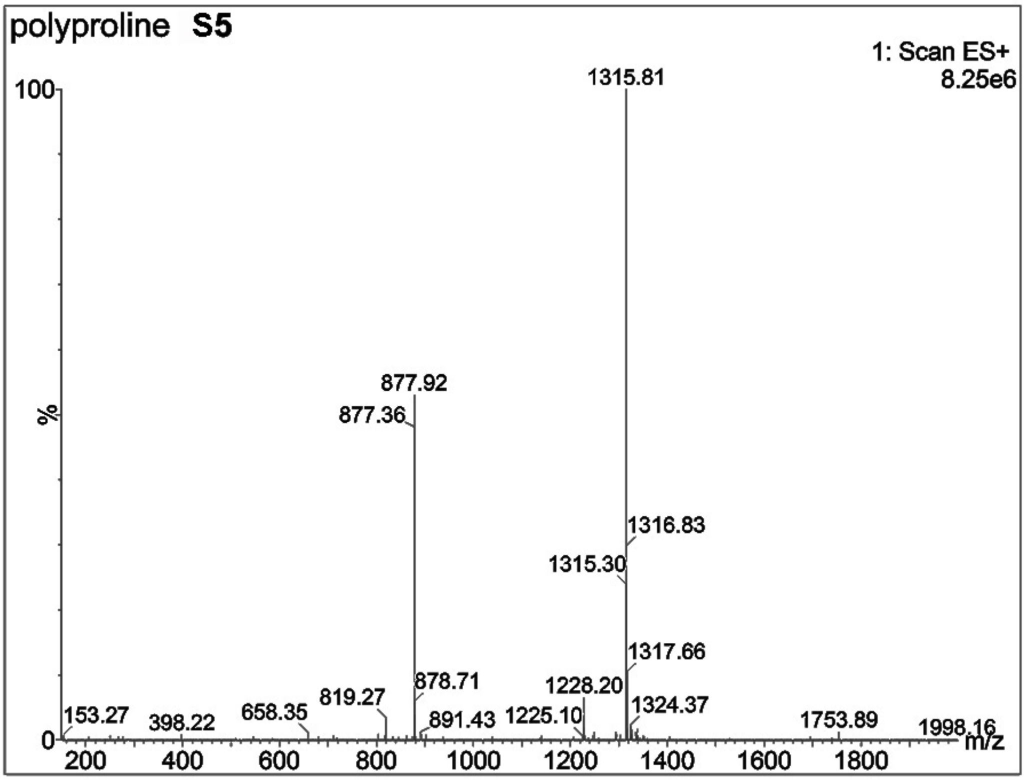

Figure S6. Analytical HPLC traces (top) and ESI mass data (bottom) of polyproline S**5**.

General procedure for the synthesis of glycopeptides via click reaction: In a typical click reaction, a small vial was charged with alkyne-functionalized CS disaccharides (total 15.6 equiv., 1.3 equiv. per azide), biotin-PEG12-azidopolyproline-OMe (1.0 equiv.), Tris[(1-benzyl-1*H*-1,2,3-triazol-4-yl)methyl]amine (TBTA, 0.3 equiv. per azide), and a small stir bar under argon atmosphere. The mixture was dissolved in degassed DMSO, and the desired amount of copper (I) iodide (0.3 mol% per azide) stock solution in DMSO and DIPEA (48 equiv.) were added. The reaction mixture was then stirred at room temperature for 7 days. The consumption of biotin-PEG12-azidopolyproline-OCH_3 was monitored by analytical reverse phase HPLC equipped with C-18 column. After the completion, anhydrous THF/MeOH (9:1) was added to give a white precipitate (TBTA and DIPEA can be removed as the glycopeptides and excess CS-disaccharides are insoluble at this condition). The white precipitate was dissolved in 200 μL of 6M aq. NaCl and purified by Sephadex G-15 column (100% H_2O) to afford desired glycopeptides as white solids upon lyophilization.

FTIR was used to monitor the azide vibrational band (~2100 cm^{-1}) for the completion of the click coupling reaction. Fourier transform infrared spectroscopy (FTIR) was carried out using a Perkin Elmer FTIR Spectrum 100 between 4000 and 800 cm^{-1} at a spectral resolution of 4 cm^{-1}, and the number of scans was 4. Samples were placed on a germanium stage and pressed before every measurement. The disappearance of the azide band in the spectra of the glycopeptides indicated the completion of the coupling reactions (Figure S7).

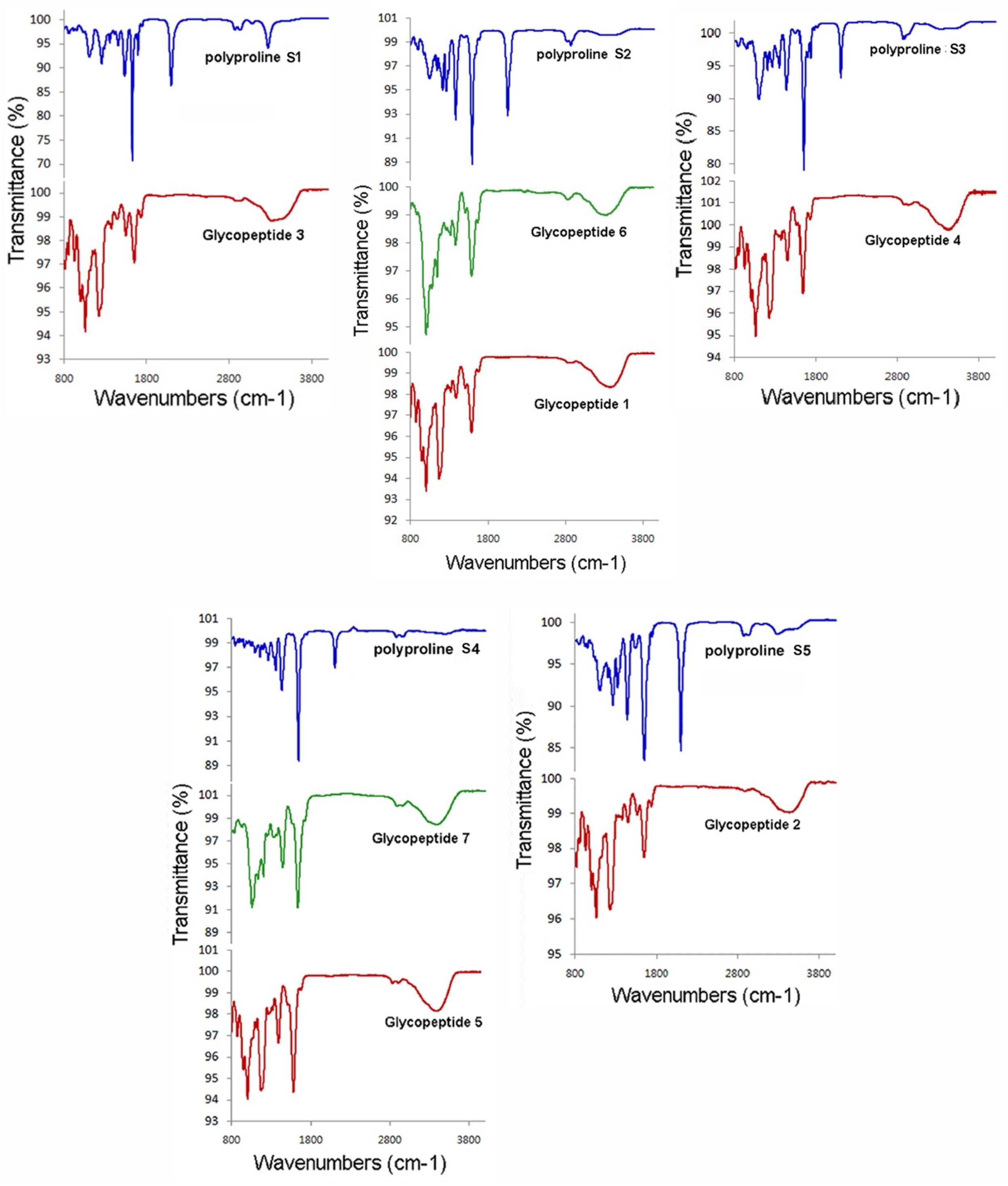

Figure S7. FT-IR spectra of polyprolines and glycopeptides.

Circular Dichroism analysis: CD spectra were recorded on an Aviv 410 circular dichroism spectrometer equipped with temperature controller. Glycopeptide solutions at concentrations of 200 μM (2.4 mM for compound **12** and **13**) were used. All sample solutions made in 10 mM sodium phosphate-dibasic buffer, pH 7.0, were equilibrated for 24 hr at 4 °C and then 1 hr at room temperature before CD measurements. Cells of 1 mm path length were used. Spectra were recorded from 260 to 190 nm at 25 °C. Mean residue ellipticity [θ] was calculated as follows;

$$[\theta] = \theta/(10 \cdot N \cdot c \cdot l)$$

θ represents the ellipticity in millidegrees, N the number of amino acid residues, c the molar concentration in mol·L^{-1}, and l the cell path length in cm.

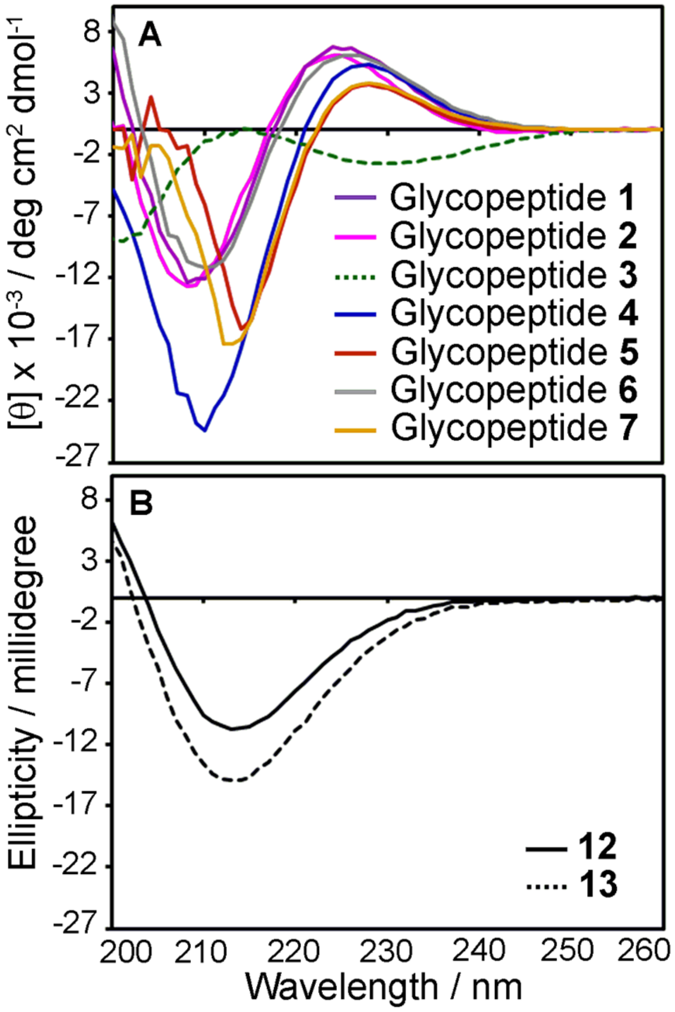

Figure S8. (A) CD spectra of **1** – **7** (200 μM) after subtraction of CD signals of corresponding CS disaccharides and normalization indicate PPII helical conformation for all polyproline-based backbones. (B) CD spectra of **12** and **13** (2.4 mM) at 25 °C.

Enzyme-Linked Immunosorbent Assay (ELISA): Pierce Streptavidin Coated High Binding Capacity plates (#15501) were rinsed 3x with PBS containing 0.05% Tween 20 (PBST) and 1x with PBS prior to the experiment. 100 μL of biotinylated glycopeptide (or CS-E polysaccharides) solutions (0.5 μM) were added to the wells and incubated overnight at room temperature. After immobilization of glycopeptides, the plates were rinsed 3x with PBST and 1xwith PBS. The surface was then blocked with 125 μL of 3% bovine serum albumin (BSA) in PBS for 1 hr, followed by rinsing with PBST and PBS. 100 μL of varying concentrations (0 nM, 10 nM, 20 nM, 30 nM, 40 nM, 50 nM and 60 nM) of NGF in PBS containing 1% BSA was added to the wells and incubated for 2 hrs at 37 °C, after which the surface was rinsed with PBST and PBS. Additional washing was done with pH 4.0 10 mM sodium acetate buffer containing 0.05% Tween 20 and 0.5 M NaCl for 10 min. The plate was then rinsed 5x with 200 μL of PBST per rinse. The plate was then washed with 10 mM TBS containing 0.05% Tween 20 (TBST) and 0.5 M NaCl for 10 min, followed by PBST and PBS. The wells were then incubated with HRP conjugated anti-NGF antibody in PBS containing 1% BSA. The absorbance signal was developed with 100 μL of TMB Substrate from Thermo Scientific Pierce (#0034021) for 20 to 30 min before quenching with 100 μL of 2 M H_2SO_4. Absorbance at 450 nm was then measured.

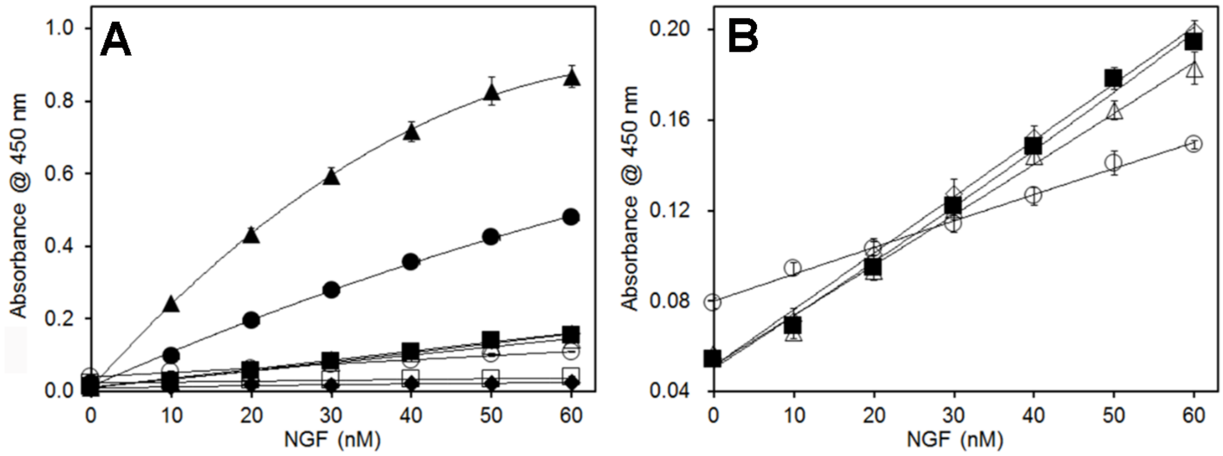

Figure S9. (A) Comparison of the binding affinities of glycopeptide **1** – **7** and CS-E polysaccharides to NGF at various concentrations. (B) Expanded view of glycopeptide **1** – **3** and CS-E polysaccharides of section a; ◊ 1, Δ 2, ■ 3, ● 4, ▲ 5, ♦ 6, □ 7, ○ CS-E polysaccharides. All data presented as mean ± SD of triplicates.

Surface Plasmon Resonance (SPR): Affinity measurements were performed using a Biacore T100 system (GE Healthcare). The CM5 sensor chip was used for all measurements. The sensor was primed with HBS-EP+ (10 mM HEPES pH 7.4, 150 mM NaCl, 3.4 mM EDTA, 0.005% P20, GE Healthcare)

buffer and was activated using amine coupling through 1:1 mixture of 0.4 M EDC and 0.1 M NHS. Streptavidin solution (0.008mg/ml) in Acetate 5.0 buffer (GE Healthcare) was used and remaining activated groups were blocked with 1 M solution of ethanolamine (pH 8.5). The final amount of streptavidin covalently immobilized on the surface was typically 700 RU. Flow cell 1 (or 3) was used as a reference to subtract nonspecific binding, drift, and the bulk refractive index, while flow cell 2 (or 4) was further immobilized with either glycopeptides or CS-E polysaccharides. Either biotinylated glycopeptides or CS-E polysaccharides (20 nM) were dissolved in HBS-EP+ buffer and were injected to flow cell 2 (or 4) at 30 μL/min until the baseline response increased by 104, 127, and 110 RU, for glycopeptide 5, 1, and CS-E polysaccharides, respectively. For a given affinity measurement, these series of peptide solutions were successively injected into the flow cells for 240 seconds of contact time and 800 seconds of dissociation time using a flow rate of 50 μL/min at 25 °C. Flow cell was regenerated using 2.5 M $MgCl_2$ at 30 μL/min. Association (k_a) and dissociation (k_d) rate constants were calculated with a 1:1 binding model using Biacore evaluation software, and K_D values were calculated from the ratio of k_d to k_a. Kinetic parameters were obtained by fitting curves to a 1:1 Langmuir model with baseline correction.

Table S3. Calculated equilibrium binding constants of NGF with **1**, **5**, or CS-E polysaccharides.

	k_a ($M^{-1}S^{-1}$)	k_d (S^{-1})	K_D (μM)[a]
CS-E poly	1.26 (±0.12) x 10^2	7.84 (±0.28) x 10^{-4}	6.21
**1**	3.46 (±0.12) x 10^2	1.24 (±0.01) x 10^{-3}	3.57
**5**	1.34 (±0.13) x 10^4	1.96 (±0.04) x 10^{-3}	0.146

[a] K_D values were obtained independently from each k_a and k_d value using Biacore T100 evaluation software v 2.0.4.

Molecular Modeling: Protein coordinates were obtained from crystal structures when available. The following PDB file was used: NGF/TrkA complex (1WWW). Protein files were downloaded from the RCSB Protein Data Bank (www.pdb.org) if available and all subsequent modifications were made using Maestro 9.6 (www.schrodinger.com). The disaccharide units were built using Maestro and a short polyproline chain was built based on X-ray crystallography structure in literature.[3] It was then lengthened by connecting multiple copies of the short fragment. Disaccharide units were then added at the appropriate locations along the polyproline chain. The chain geometry was fixed, and the disaccharide side chain conformation was energy minimized using OPLS_2005 until the rms deviation was less than 0.1 Å individually.

To determine the binding site, the NGF/TrkA complex was first loaded from the indicated PDB file[4] and the water molecules removed for simplicity. Coarse binding sites were first identified by examining positively charged residues on the NGF/TrkA complex. Rigid body docking of the glycopeptide was then performed manually and at least Arg314 or Arg342 on TrkA was included in the binding site. The glycopeptide was brought close to the positively charged residues such that the sugar pendants were within 3Å of the positive residue while avoiding steric clashes. Bonds in the disaccharide units and interacting residues on NGF/TrkA were rotated to maximized potential interactions and the energy was briefly minimized as described above. Potential hydrogen bonding partners on NGF/TrkA were then identified. This was done by first identifying residues in the vicinity of the sugar pendants that can participate in hydrogen bonding (Ser, Asp, Glu, Thr, Tyr). The side groups of these residues and the sugar pendants were then rotated to bring them into optimal hydrogen bonding distance (2 – 4 Å) and bond angle (~170 to 180° between O-H-O). If these manipulations failed, hydrogen bonding between identified residue and sugar pendant was deemed unlikely and any structural changes were undone. Large manipulations to sugar pendants and participating residues were eschewed as much as possible to avoid potential disruptions to electrostatic interactions identified above. Energies of the interacting residues and interacting sugar pendants were then minimized.

Table S4. Predicted glycopeptide binding sites on the NGF/TrkA complex. Bold indicates binding residues for electrostatic interactions; Non-bolded indicates binding residues for hydrogen bonding.

Glycopeptide	Binding Residues (TrkA)[a]	Binding Residues (NGF: Monomer A)[b]	Binding Residues (NGF: Monomer B)[b]
**1**	Glu334, **Arg342**	**Arg9**	Asp16, **Lys34,** Glu35, **Lys57,** Asp60, **Arg69, Lys 74, Arg103, Lys 115**
**2**	**Arg342**	**Arg9**	**Lys34,** Glu35, **Lys57,** Asp60, **Arg69, Lys 74, Arg103, Lys 115**
**3**	**Arg314, Arg342**	**Arg9**	**Lys34, Lys57, Arg69, Lys 74, Lys 115**
**4**	Ser312, **Arg314,** Glu331, **Arg342**	Glu11	Ser3, Asp16, Glu35, **Lys57, Arg5, Arg69,** Ser113, **Lys115**
**5**	Ser312, **Arg314,** Glu324, Glu331, Glu334, **Arg342**	**Arg9**	Ser73, **Lys74, Lys115**

[a] One or two basic residues on the TrkA component participate in binding with each glycopeptide.

[b] At least three basic residues on the NGF component participate in binding with each glycopeptide.

Cellular Assays. PC12 cells were maintained in T75 tissue culture flasks in RPMI 1640 medium (Gibco 21870-076) containing 10% heat-inactivated horse serum (HI-HS), 5% fetal bovine serum (FBS) and 1% penicillin/streptomycin. Stock cultures from liquid nitrogen were grown at 37°C with 5% CO_2 in a humidified chamber for 72 hr before experiments. For all experiments, cells were seeded on 13 mm round glass coverslips (Paul Marienfeld 0111530). The coverslips were treated with 65% nitric acid for three days, washed with distilled water, 70% ethanol and 100% ethanol 3x each for 30 minutes with gentle rocking and were dried in the cell culture hood overnight with UV sterilization. For cell attachment, the coverslips were coated with laminin (25 μg/mL in PBS; Sigma L2020) at 37 °C for 2 hr and washed 3x with PBS before use. For neurite outgrowth assay, cells were seeded at a density of 100 cells/mm^2 in RPMI 1640 differentiation medium containing 1% HI-HS. Cells were allowed to attach to the surface for 1 hr at 37 °C and incubated with fresh differentiation medium in 24-well plate for another hour. Glycopeptides (10 μM) or CS-E polysaccharides (Seikagaku AMS.NACS-E2.SqC.10) were incubated

with 4 ng/mL NGF (PeproTech 450-01) in differentiation medium for 1hr at room temperature before adding to the cells together. CS-E polysaccharides have a molecular weight of ~60 kD and contain ~60% CS-E disaccharide units. 2 μM of CS-E polysaccharides was used to maintain the same amount of CS-E disaccharide units as the glycopeptides. After 3 days, the cells were fixed with formalin solution (Sigma HT5012) for 15 minutes at room temperature and rinsed 2x with PBS. Bright-field images were taken using Olympus IX71 Inverted Microscope at 20x magnification. For each treatment, 400 - 500 randomly selected single cells were counted. The percentage of neurite-bearing cells was determined by counting the number of cells with visible neurites. All experiments were repeated three times and done in duplicate each time.

Western blot. PC12 cells were seeded at a density of 300 cells/mm^2 and one coverslip was used for each treatment (~40,000 cells). Cells were starved in differentiation medium for 12 - 18 hr before use. For glycopeptide treatment, cells were incubated with fresh differentiation medium containing 10 μM glycopeptide for 1 hr at 37 °C. To induce TrkA phosphorylation, cells were treated with 4 ng/mL NGF for 5 minutes at 37°C. Cells were washed once with ice cold PBS and lysed using RIPA lysis buffer (Santa Cruz Biotechnology SC-24948) containing protease and phosphatase inhibitors (Thermo Scientific 88668). The lysates were concentrated and purified by overnight ethanol precipitation at -20 °C. Protein pellets were obtained by centrifuging at 14,000 g for 30 minutes in a 4 °C pre-cooled centrifuge. The pellets were dissolved in 1x LDS sample buffer (Life Technologies NP0008), separated on 4 - 12 % SDS-PAGE gel (Life Technologies NP0321BOX) and transferred to nitrocellulose membrane. Membranes were blocked with 5% milk in TBST (for total TrkA) or 5% BSA in TBST (for pTrkA) for 1 hr at room temperature and probed with anti-TrkA (763) rabbit antibody (1:1500; Santa Cruz Biotechnology SC-118) and anti-pTrkA (Tyr490) rabbit antibody (1:1500; Cell Signaling 9141L) overnight at 4 °C, followed by 2 hr incubation with horseradish peroxidase-conjugated anti-rabbit IgG antibody (Promega W4011). Enhanced chemiluminescence system (GE Healthcare RPN 2232) was used for detection and images were captured using Bio-Rad ChemiDoc™ MP imaging system. The bands were quantified using ImageJ

(http://imagej.nih.gov/ij/) and the ratio of phospho-TrkA to total TrkA was calculated and expressed as arbitrary units (a.u.).

References

1. F. Bélot, J.-C. Jacquinet, *Carbohydr. Res.,* **2000**, *326*, 88.

2. H. Lucas, J. E. M. Basten, T. G. van Dinther, D. G. Meuleman, S. F. van Aelst, and C. A. A. van Boeckel, *Tetrahedron*, **1990**, *46,* 8207.

3. P.M. Cowan, S. McGavin, *Nature,* **1995**, *176*, 501.

4. C. Wiesmann, M.H. Ultsch, S.H. Bass, & A.M. de Vos, *Nature,* **1999**, *401*, 184.

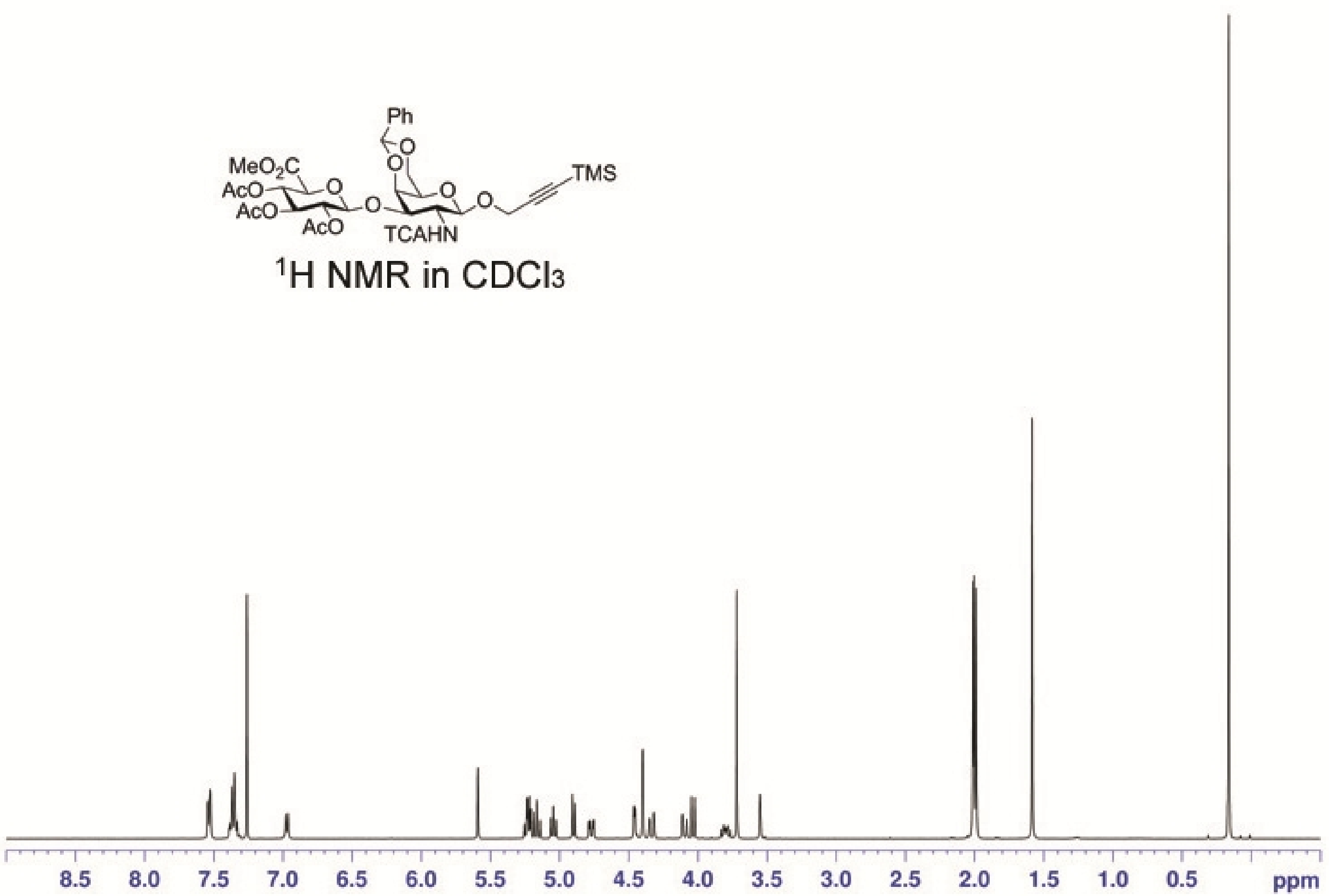
Ph
MeO2C
AcO
AcO
AcO
TCAHN
TMS
^{1}H NMR in CDCl3
8.5 8.0 7.5 7.0 6.5 6.0 5.5 5.0 4.5 4.0 3.5 3.0 2.5 2.0 1.5 1.0 0.5 ppm

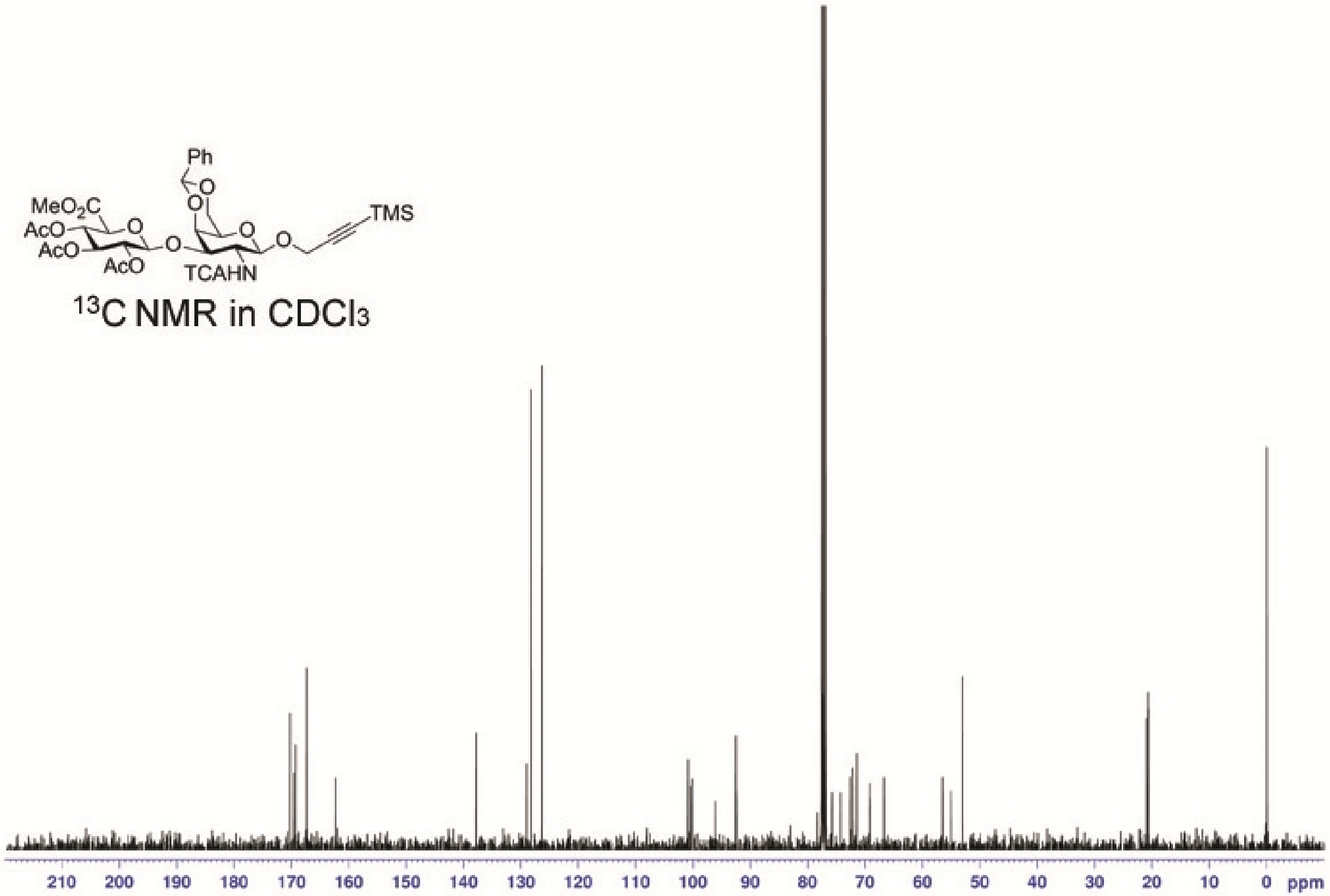

Ph
MeO2C
AcO
AcO
AcO
TCAHN
TMS
13C NMR in CDCl3
210 200 190 180 170 160 150 140 130 120 110 100 90 80 70 60 50 40 30 20 10 0 ppm

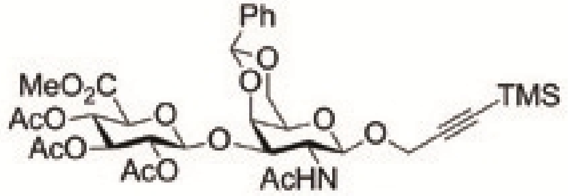

^{1}H NMR in $CDCl_3$

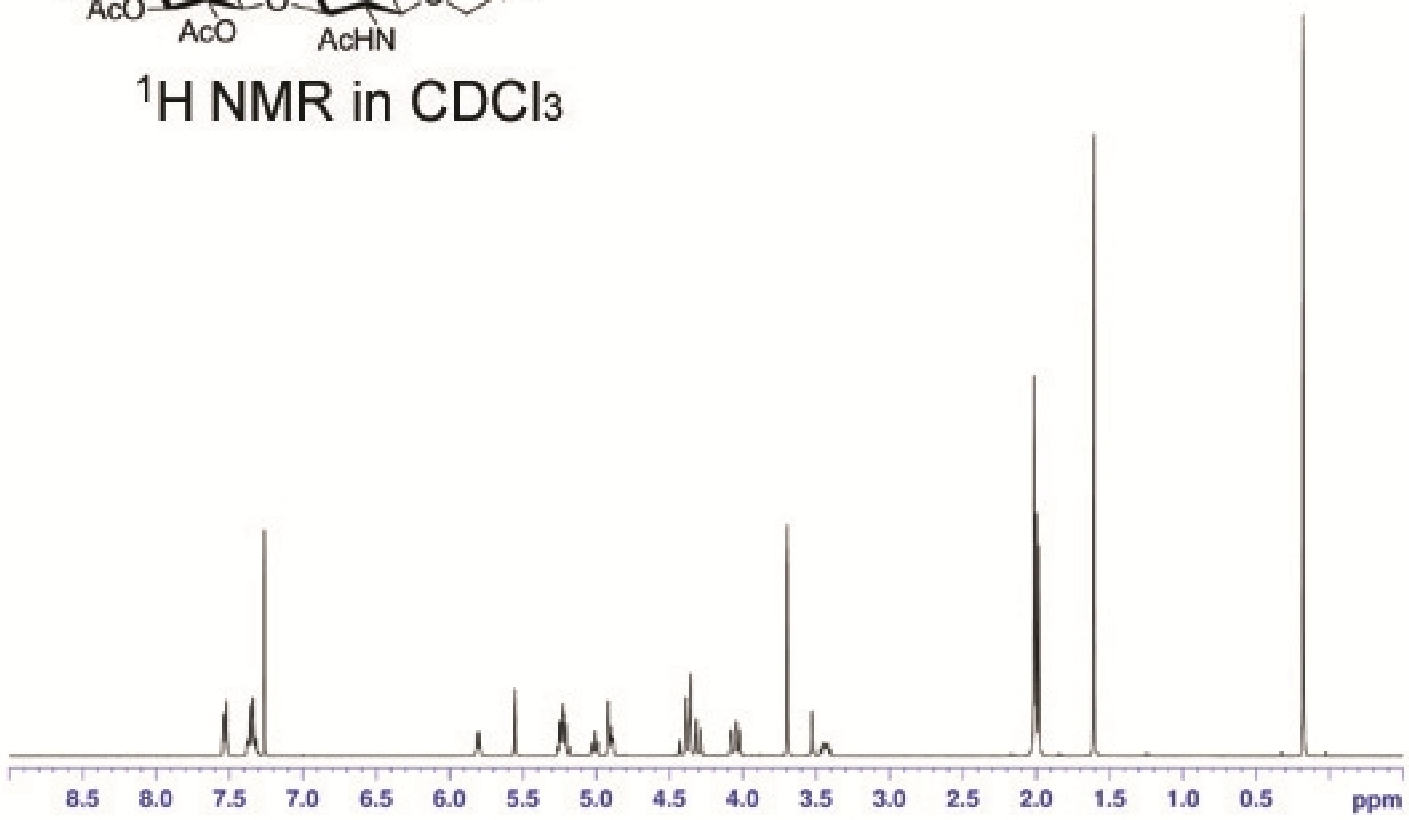

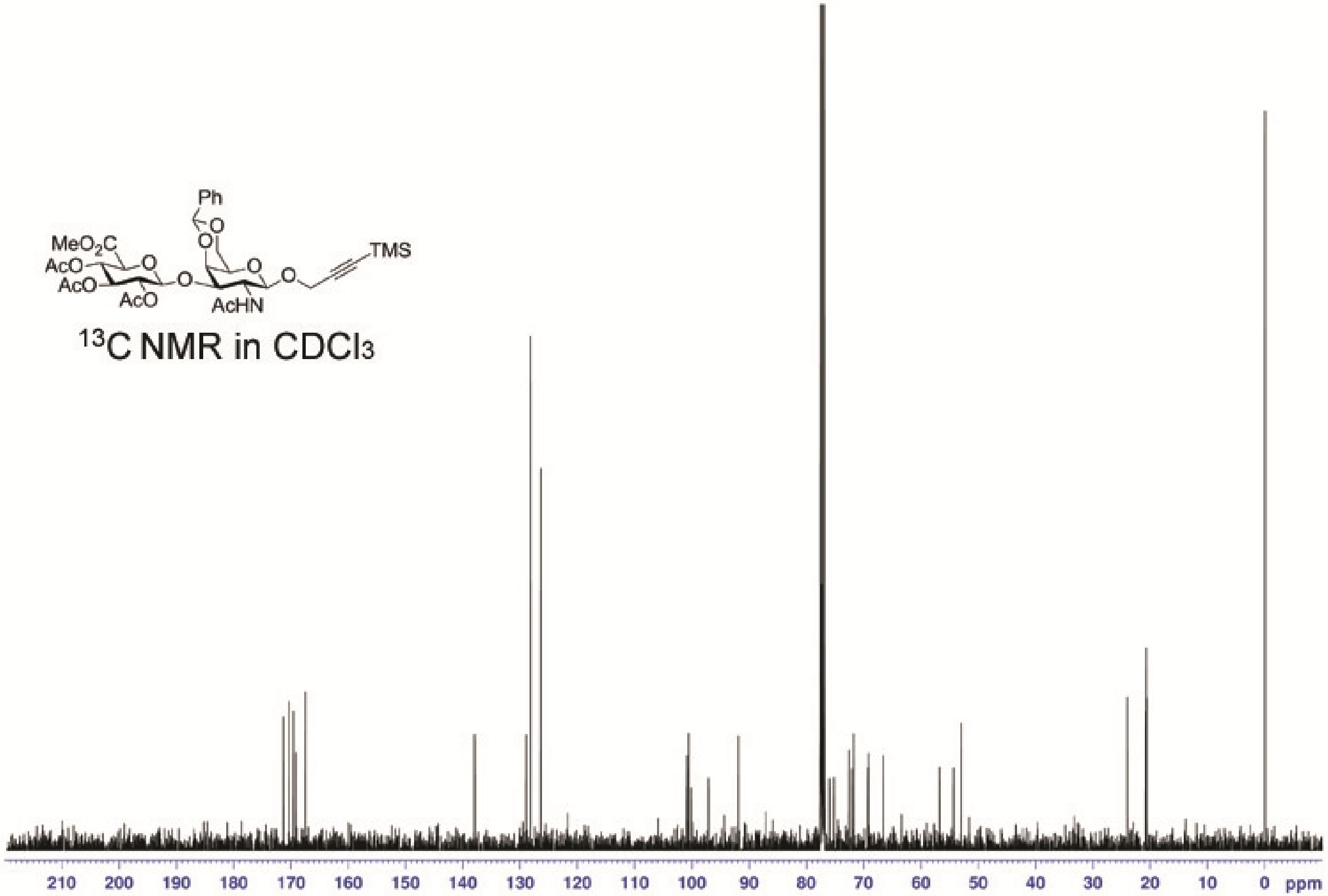

^{13}C NMR in $CDCl_3$

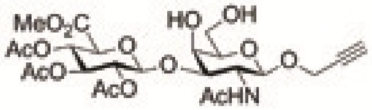

^{1}H NMR in CD_3OD

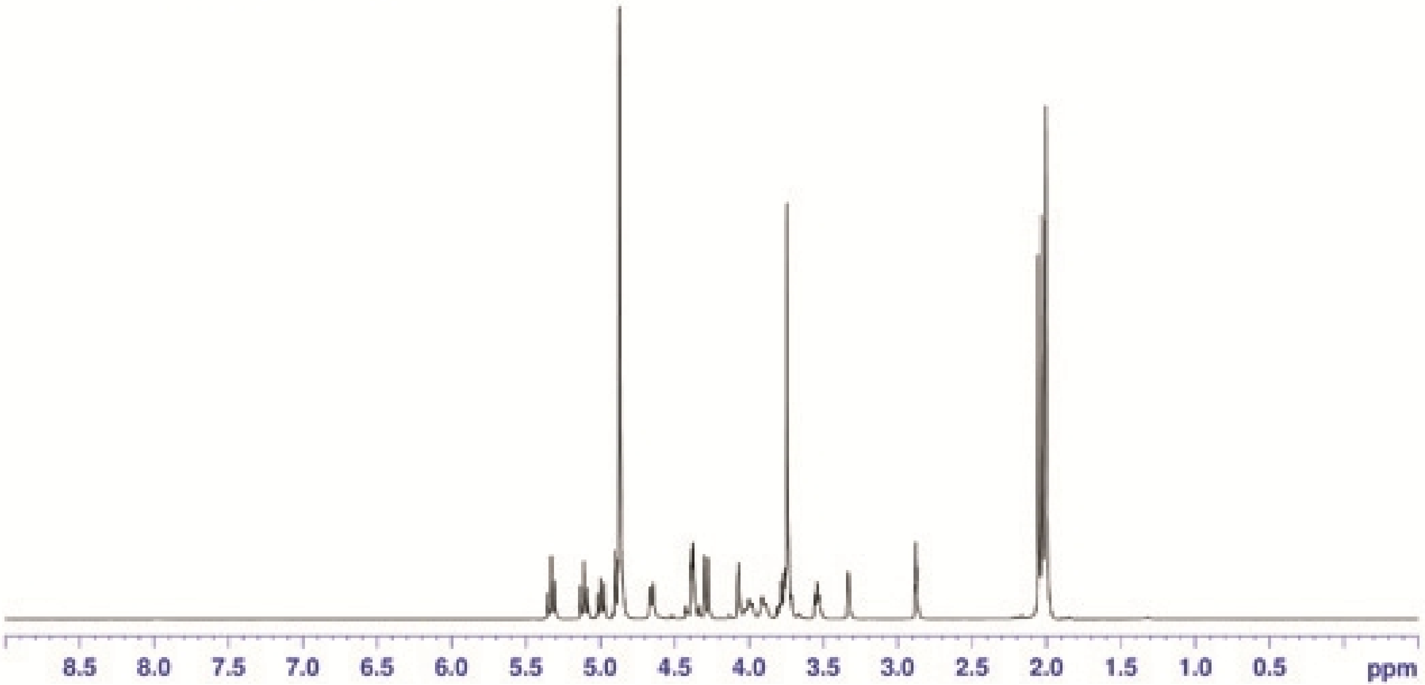

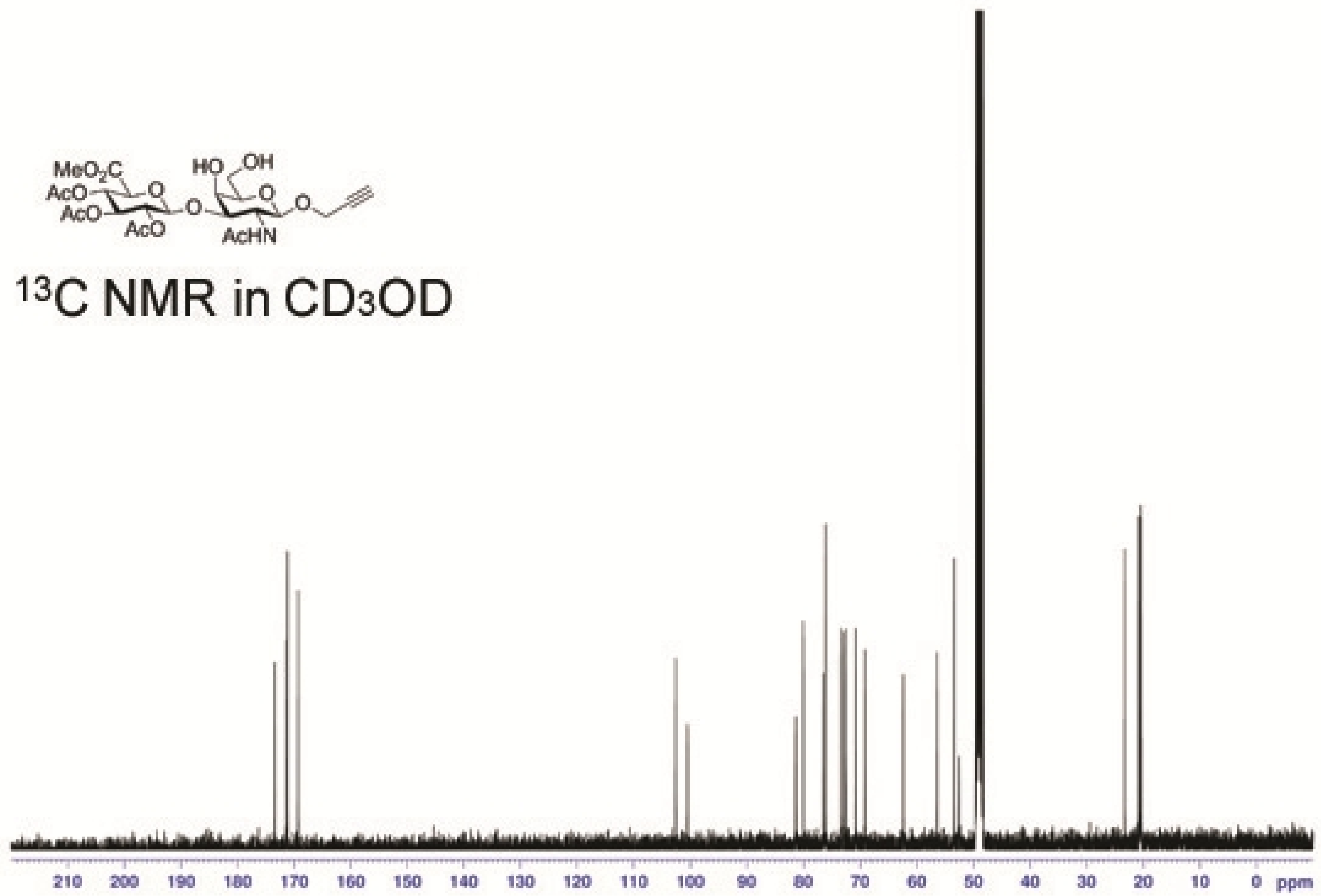
MeO2C
AcO
AcO
AcO
HO
OH
AcHN
13C NMR in CD3OD
210 200 190 180 170 160 150 140 130 120 110 100 90 80 70 60 50 40 30 20 10 0 ppm

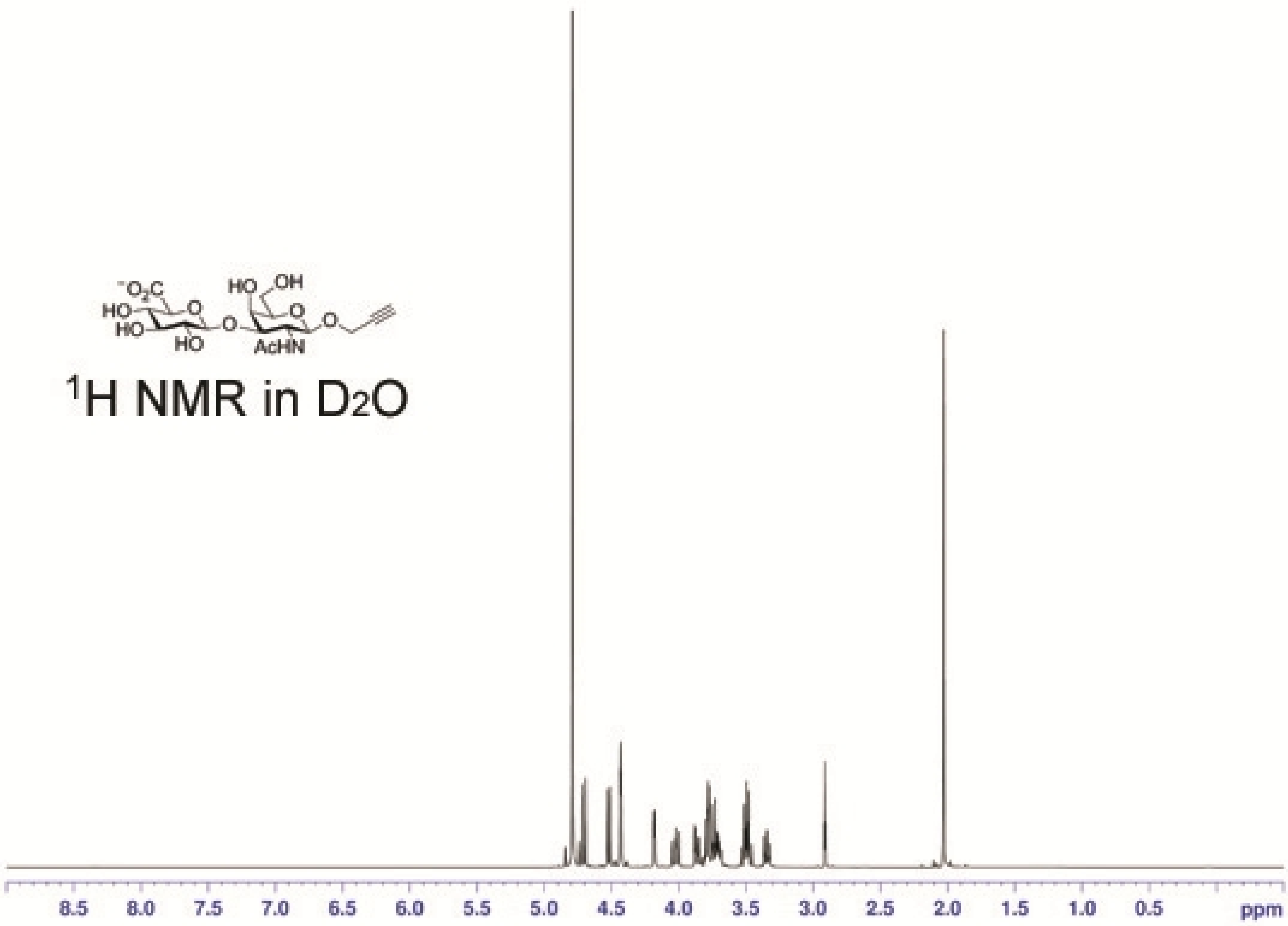

⁻O2C
HO
HO
HO
O
O
HO OH
O
AcHN
O
1H NMR in D2O
8.5 8.0 7.5 7.0 6.5 6.0 5.5 5.0 4.5 4.0 3.5 3.0 2.5 2.0 1.5 1.0 0.5
ppm

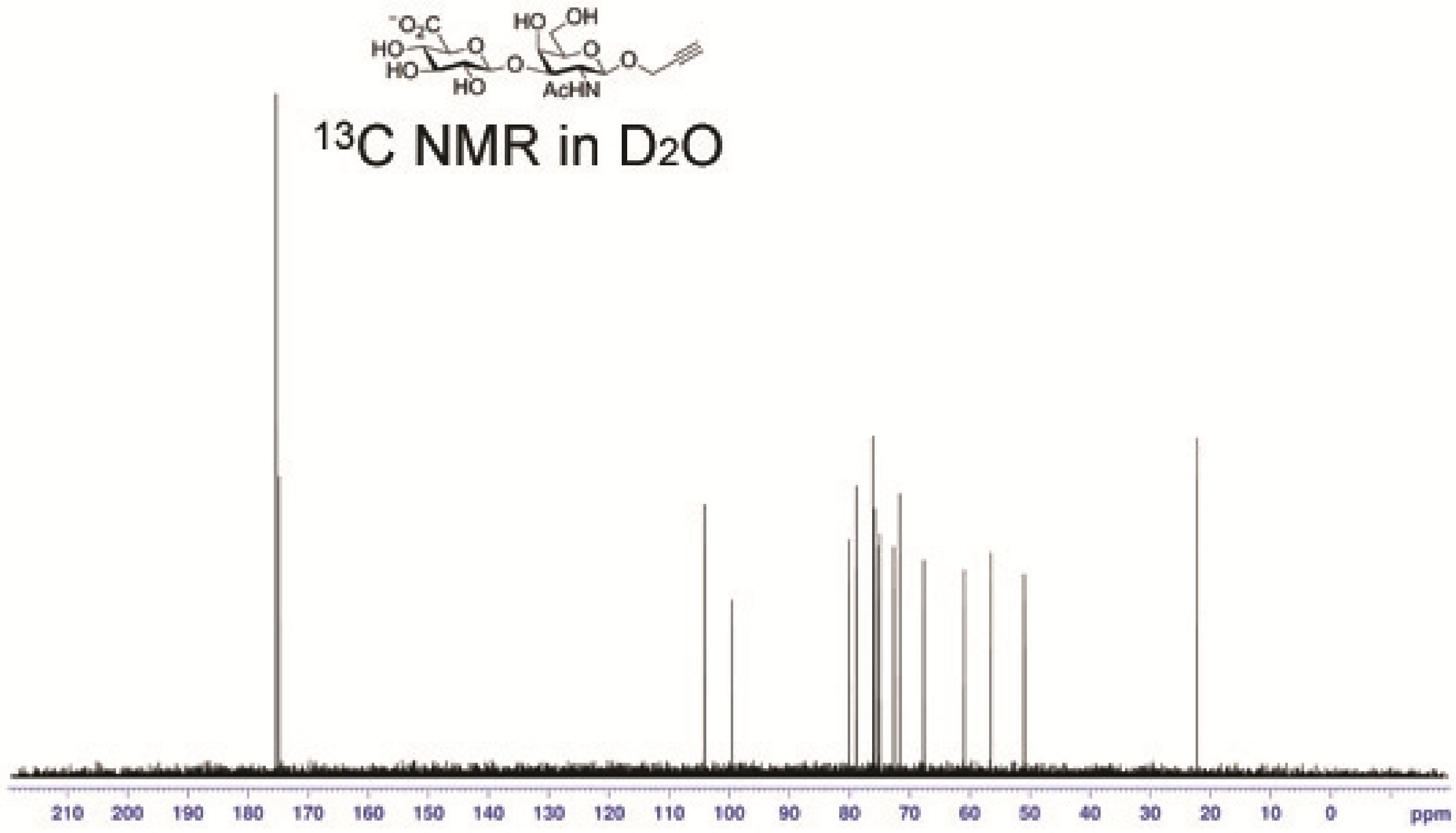

$^{-}O_2C$
HO
HO
HO
HO
OH
AcHN
13C NMR in D2O
210 200 190 180 170 160 150 140 130 120 110 100 90 80 70 60 50 40 30 20 10 0
ppm

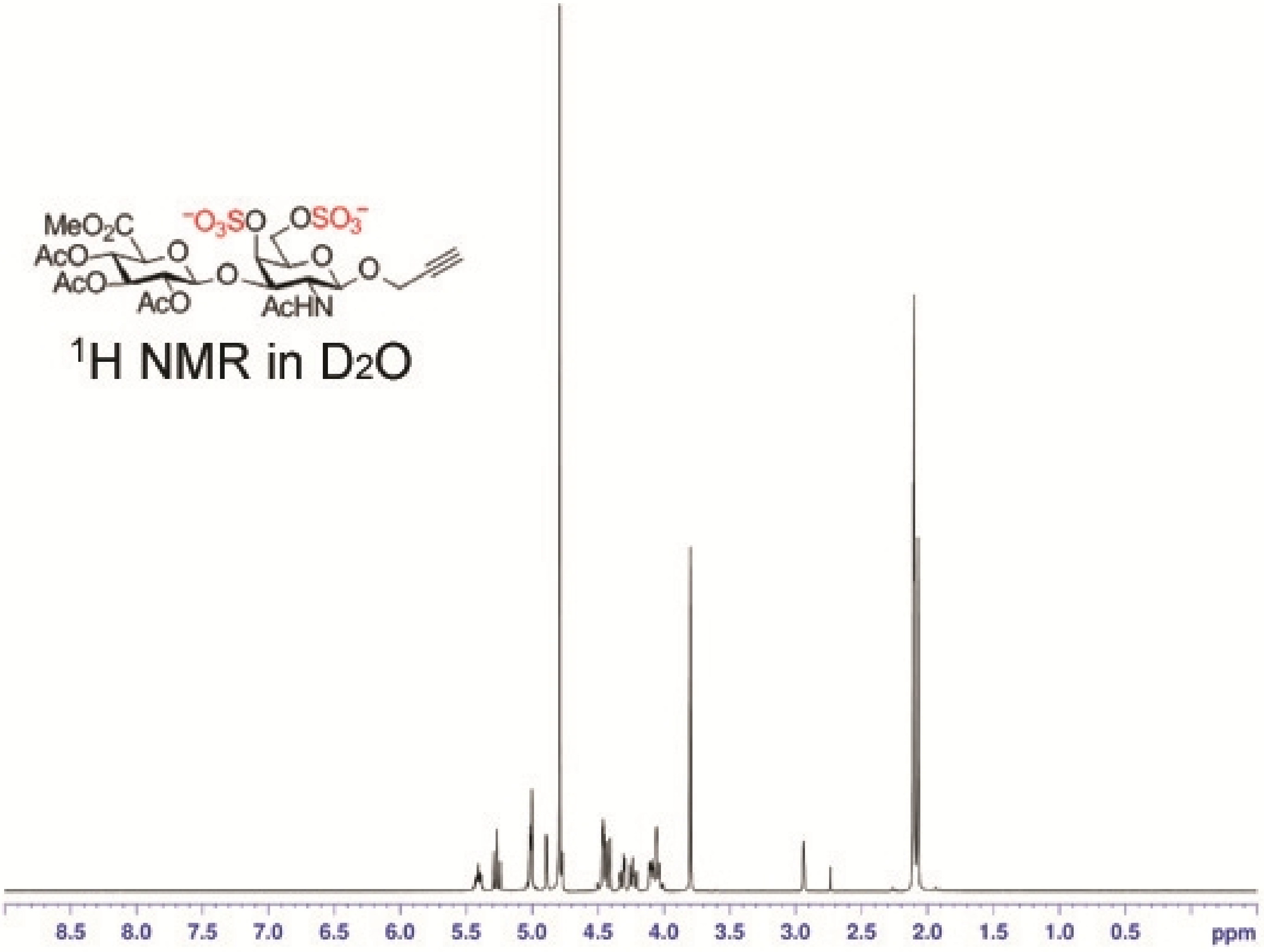
MeO2C
AcO
AcO
AcO
−O3SO
OSO3−
AcHN
1H NMR in D2O
8.5
8.0
7.5
7.0
6.5
6.0
5.5
5.0
4.5
4.0
3.5
3.0
2.5
2.0
1.5
1.0
0.5
ppm

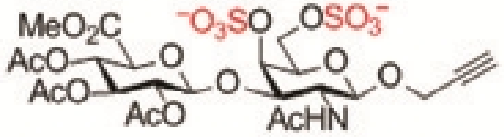

^{13}C NMR in D_2O

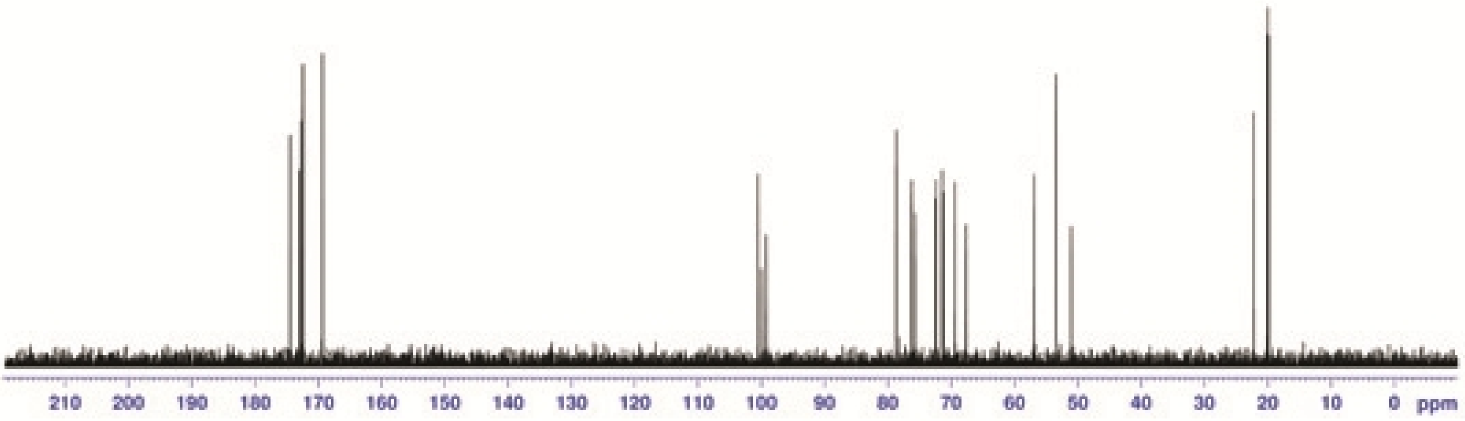

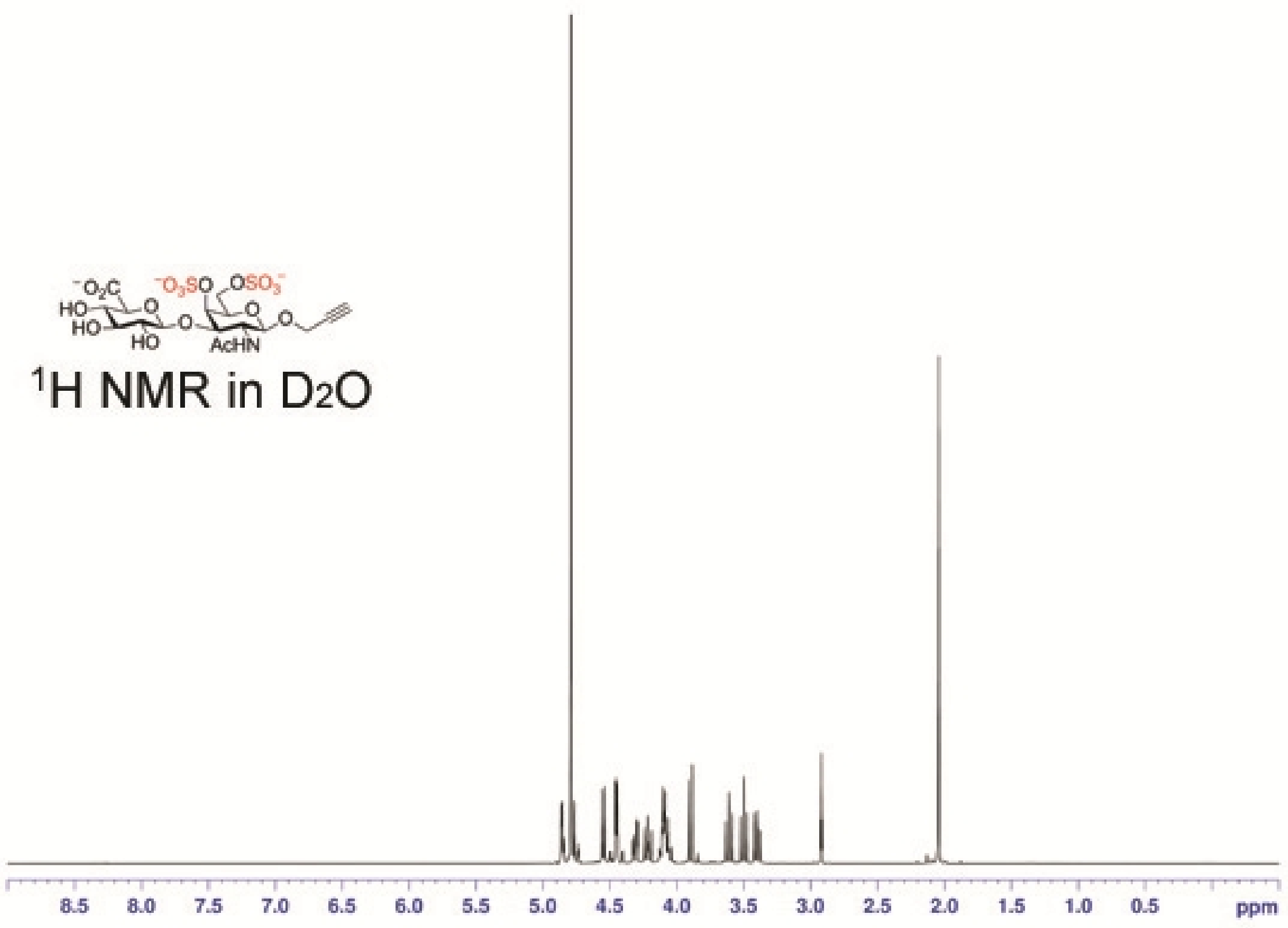

^{1}H NMR in D2O
8.5 8.0 7.5 7.0 6.5 6.0 5.5 5.0 4.5 4.0 3.5 3.0 2.5 2.0 1.5 1.0 0.5 ppm

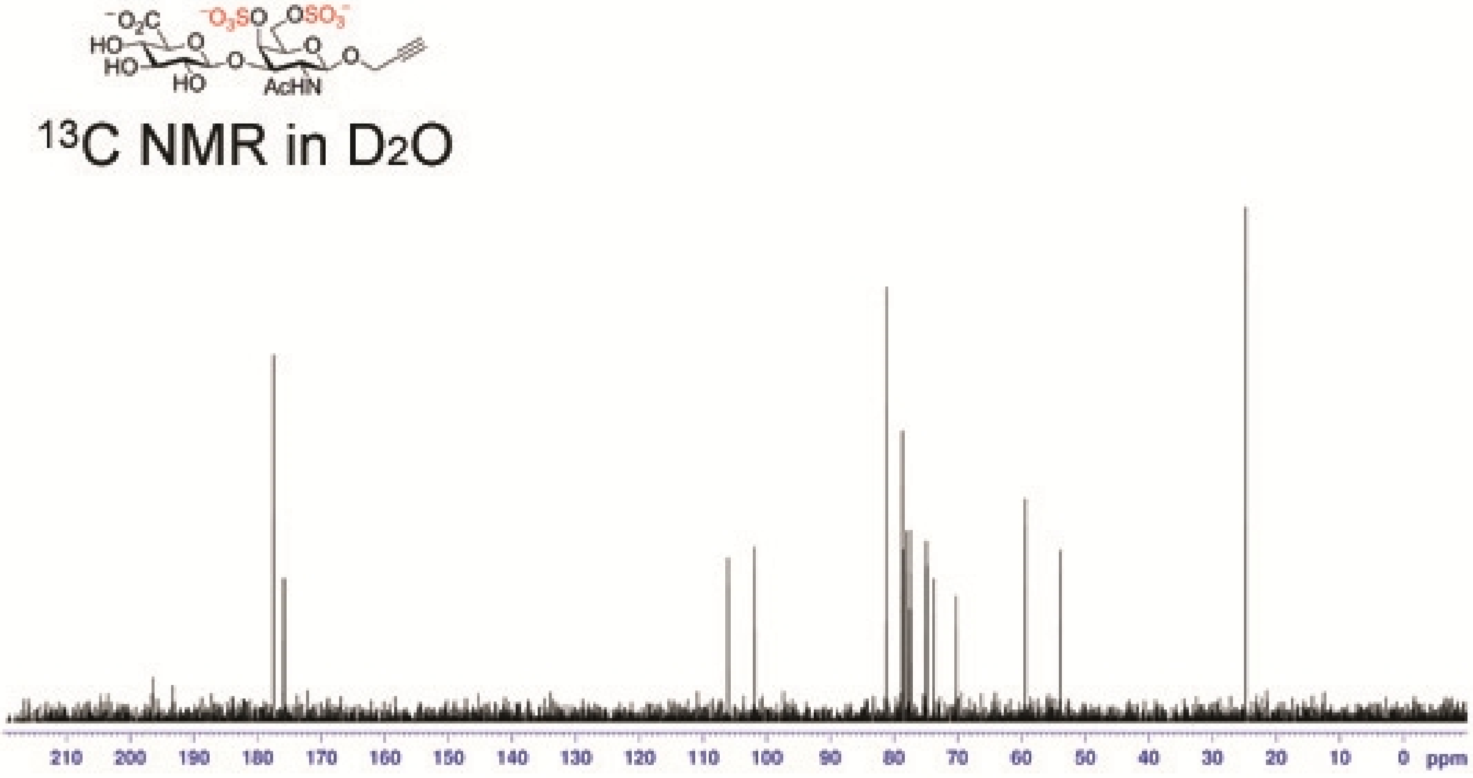
13C NMR in D2O
210 200 190 180 170 160 150 140 130 120 110 100 90 80 70 60 50 40 30 20 10 0 ppm

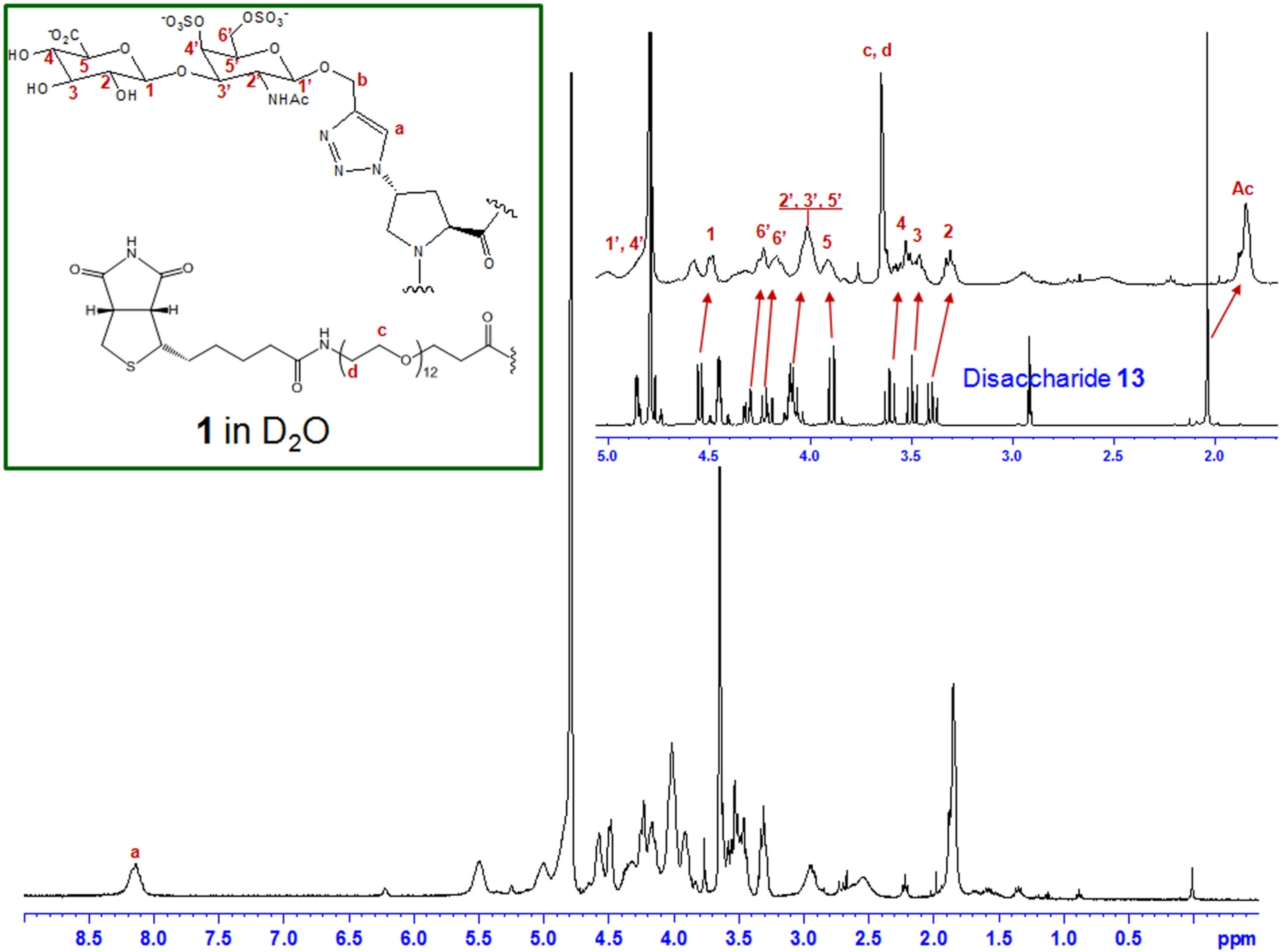
1 in D$_2$O
Disaccharide 13
1', 4'
1
6'
6'
2', 3', 5'
5
c, d
4
3
2
Ac
a
ppm

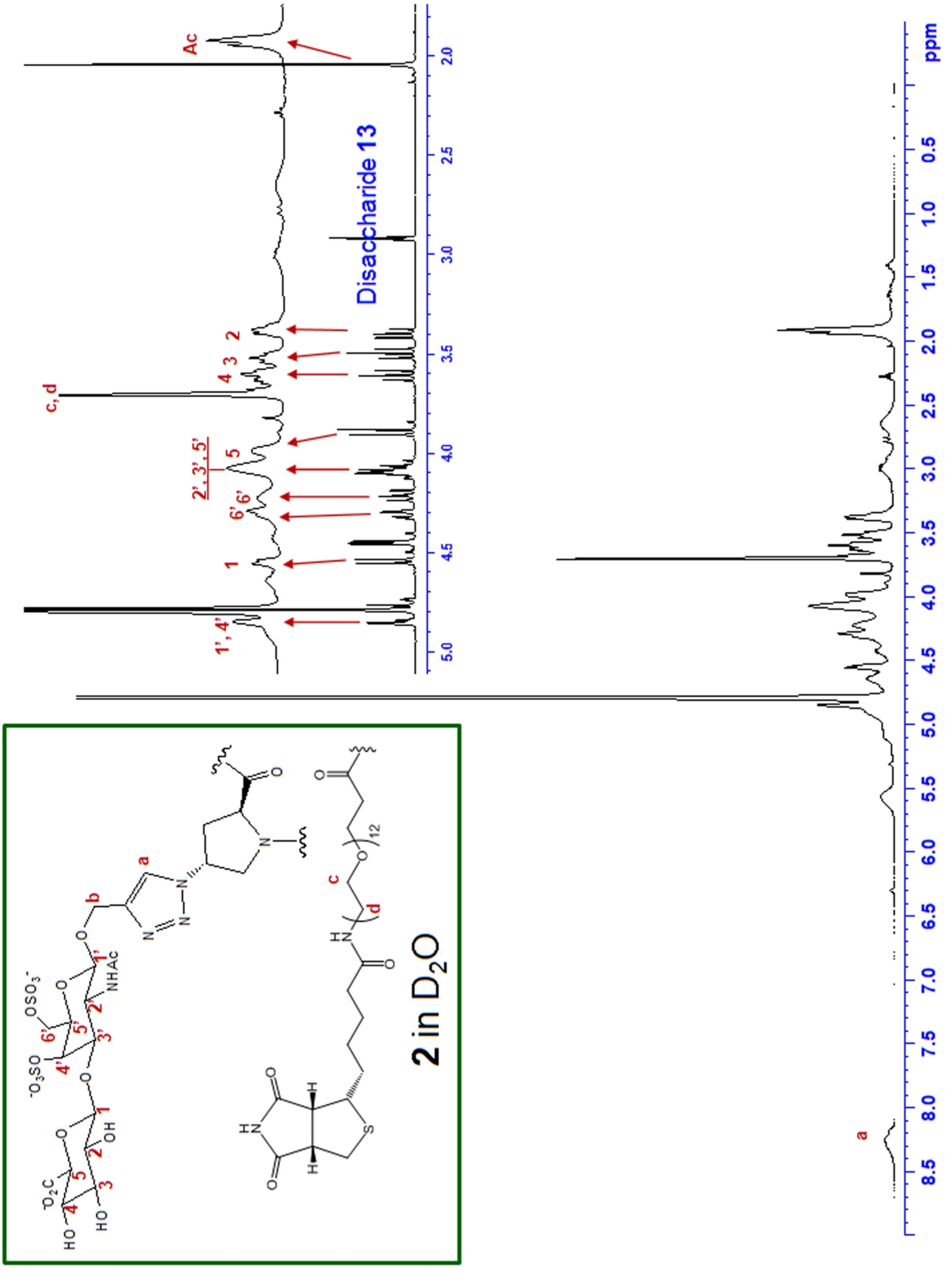

2 in D$_2$O
Disaccharide 13
Ac
c, d
ppm

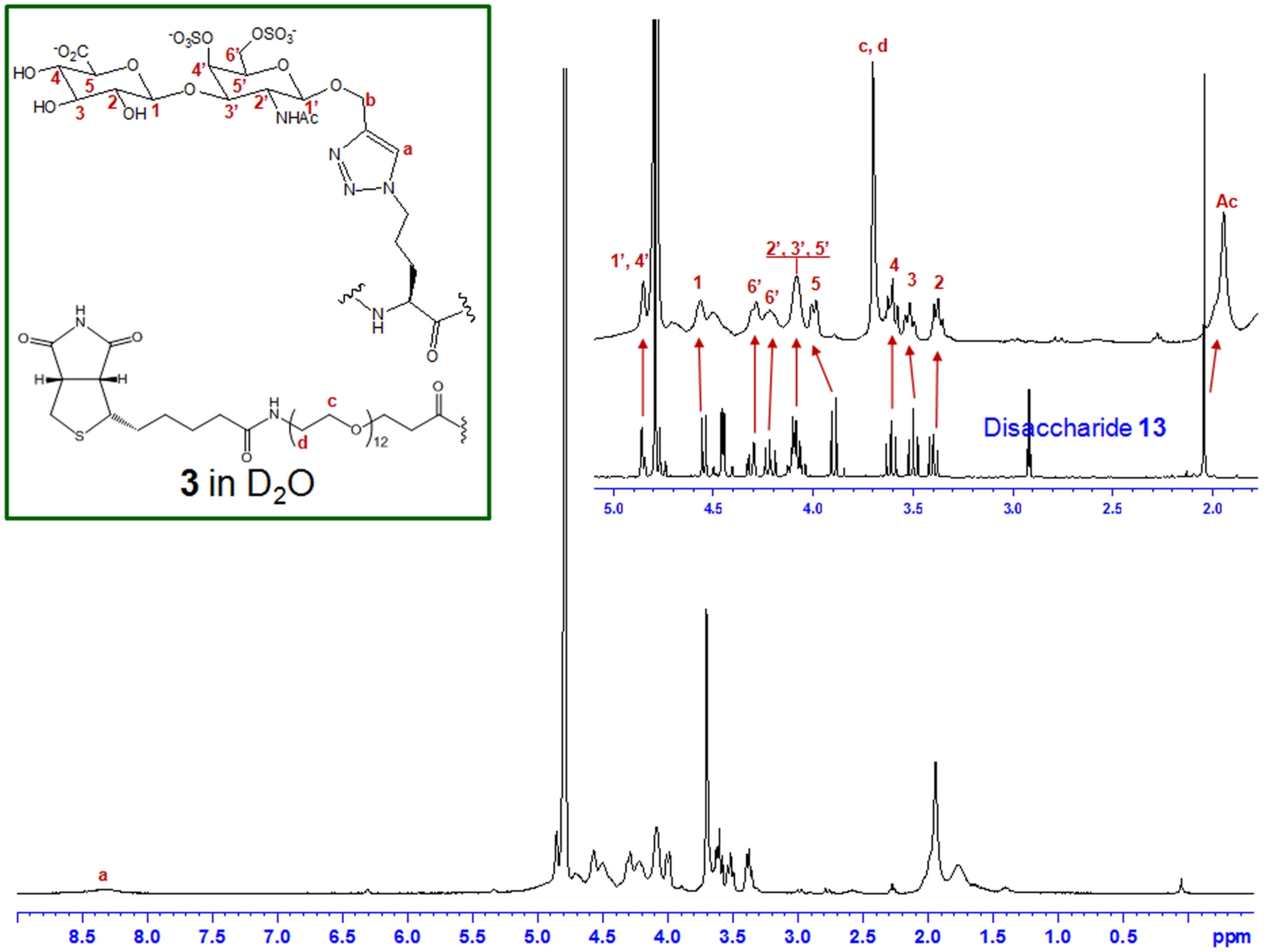

3 in D2O
Disaccharide 13
1', 4'
1
6'
6'
2', 3', 5'
5
c, d
4
3
2
Ac
a
b
c
d
NHAc
ppm

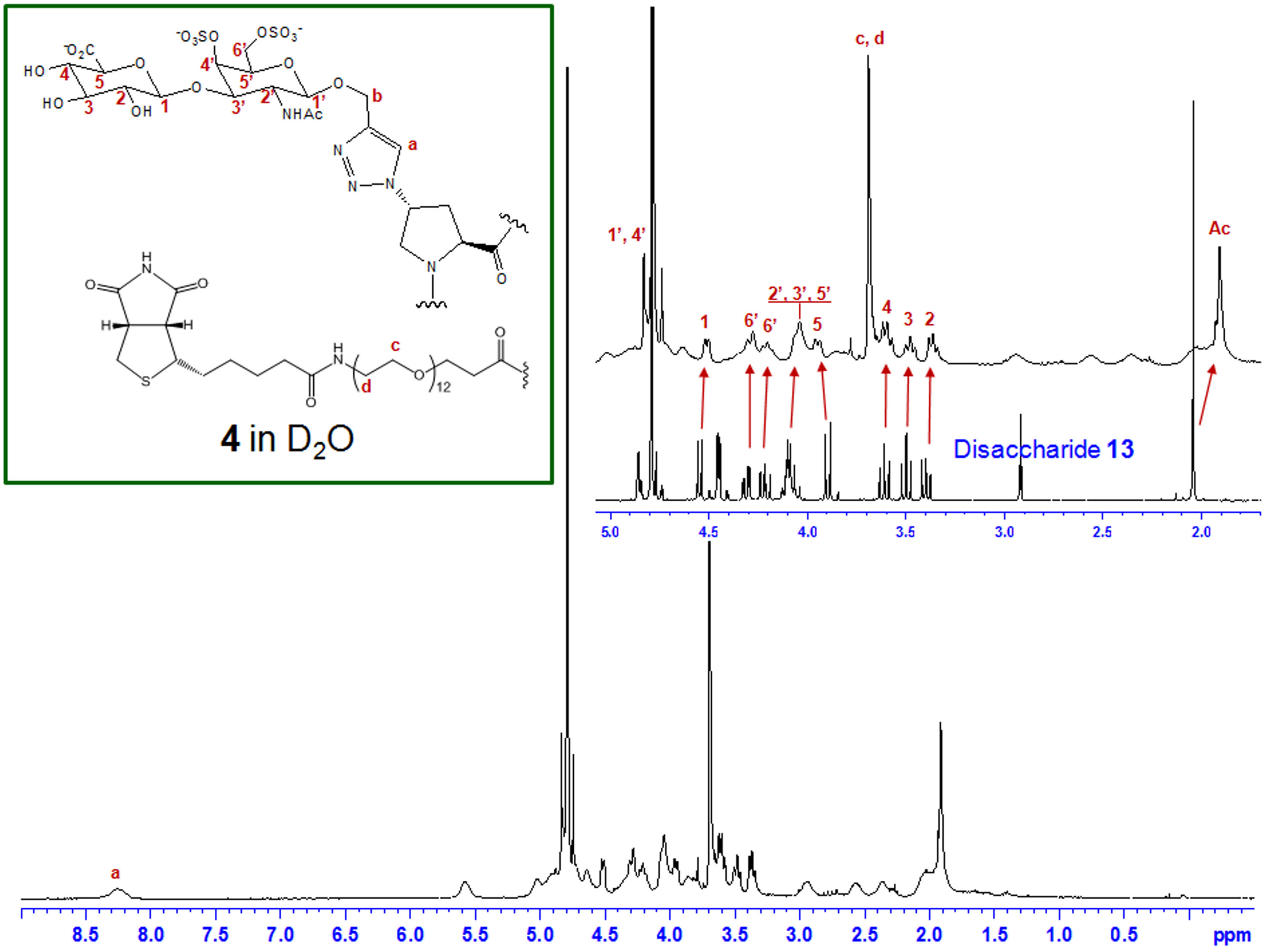
4 in D_2O
1', 4'
1
6'
6'
2', 3', 5'
5
c, d
4
3
2
Ac
Disaccharide 13
a
ppm

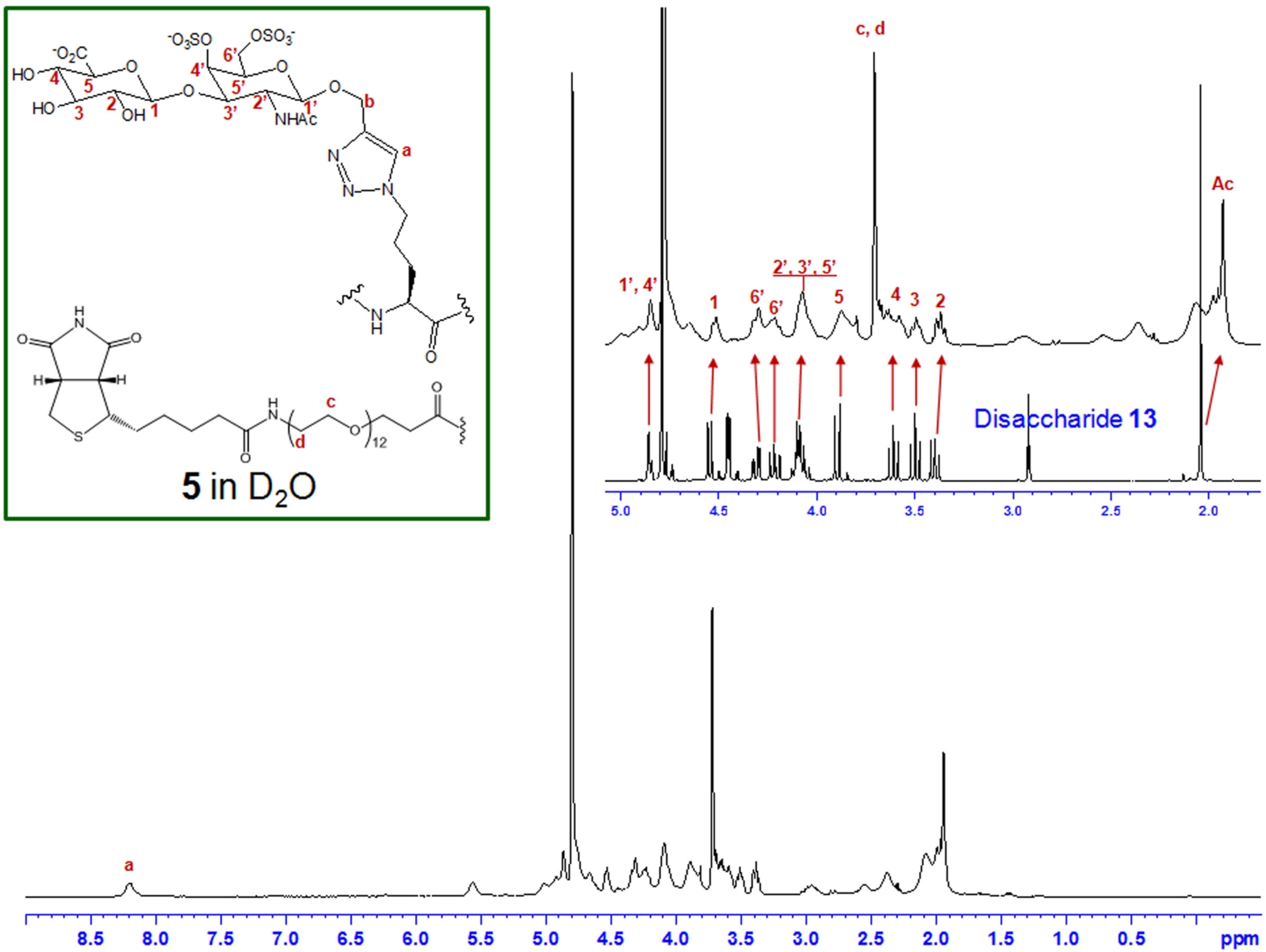
5 in D2O
Disaccharide 13
c, d
Ac
1', 4'
1
6'
6'
2', 3', 5'
5
4
3
2
a
ppm

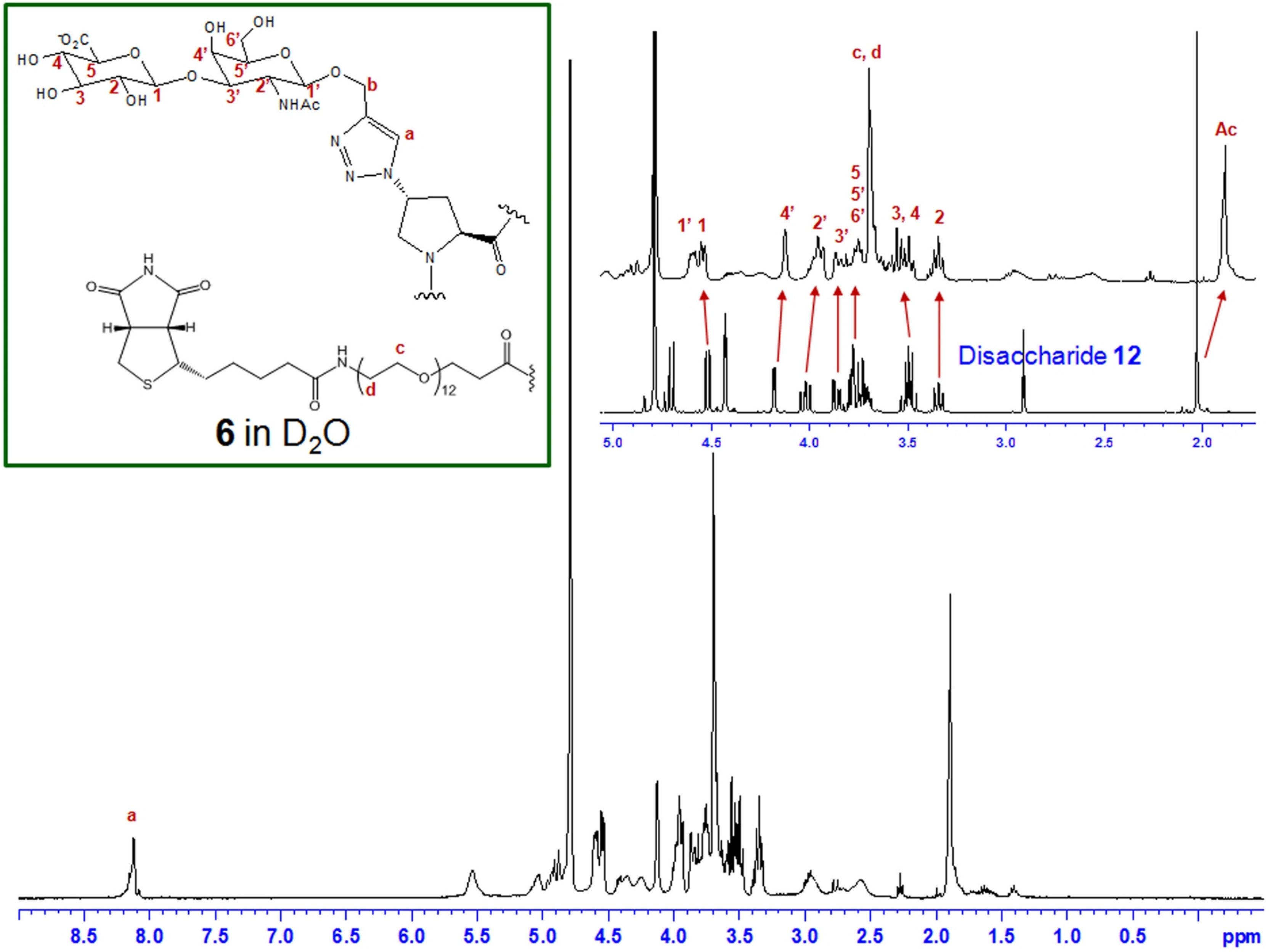

6 in D_2O
Disaccharide 12
a
b
c
d
c, d
Ac
1' 1
4'
2'
3'
5
5'
6'
3, 4
2
ppm

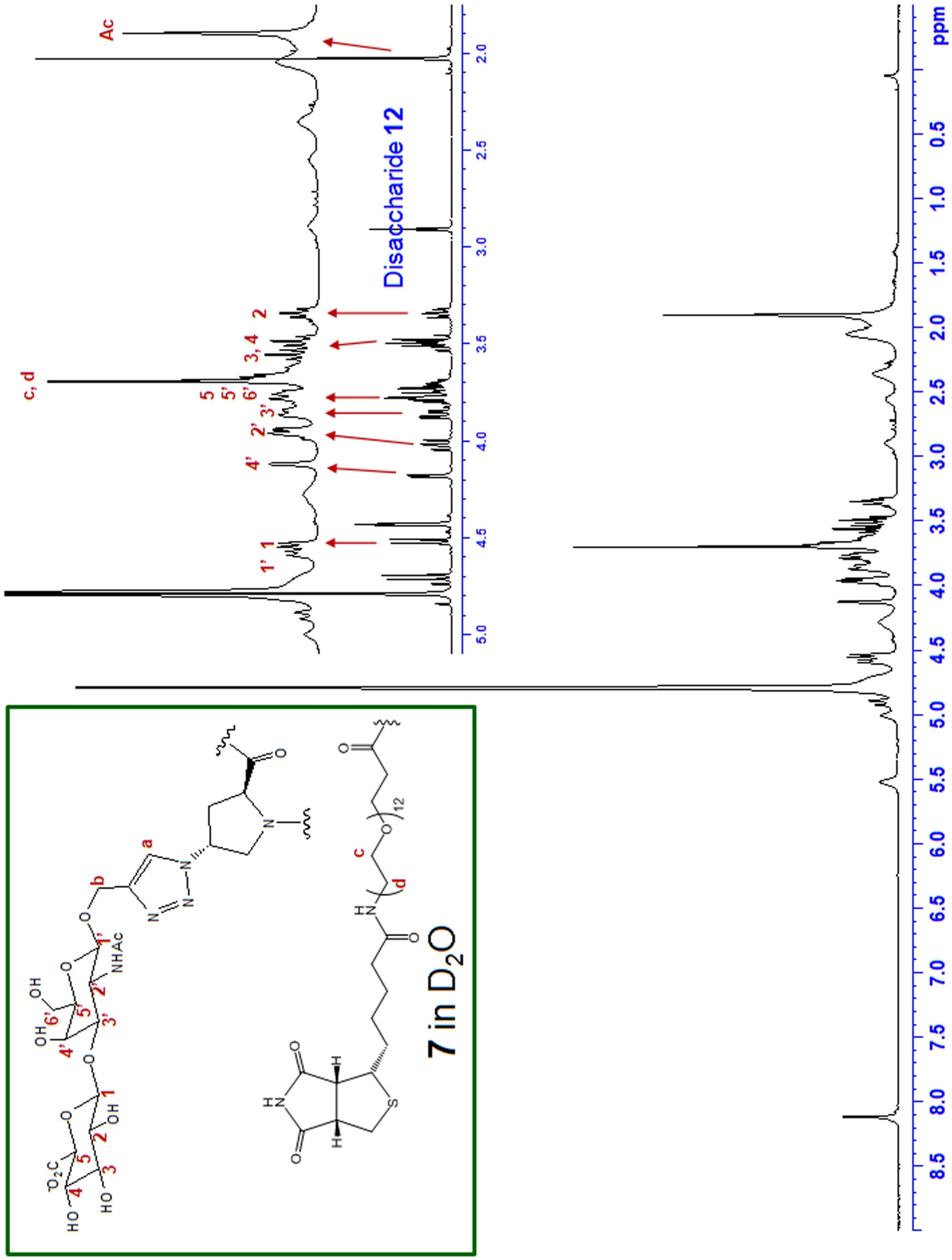

Ac
c, d
2
3, 4
5
5'
6'
3'
2'
4'
1' 1
Disaccharide 12
ppm
NHAc
a
b
c
d
12
7 in D2O
